# Timing of antipsychotics and benzodiazepine initiation during a first episode of psychosis impacts clinical outcomes: Electronic health record cohort study

**DOI:** 10.3389/fpsyt.2022.976035

**Published:** 2022-09-23

**Authors:** Maite Arribas, Marco Solmi, Trevor Thompson, Dominic Oliver, Paolo Fusar-Poli

**Affiliations:** ^1^Early Psychosis: Interventions and Clinical-Detection (EPIC) Lab, Department of Psychosis Studies, Institute of Psychiatry, Psychology and Neuroscience, King’s College London, London, United Kingdom; ^2^Department of Psychiatry, University of Ottawa, Ottawa, ON, Canada; ^3^Department of Mental Health, The Ottawa Hospital, Ottawa, ON, Canada; ^4^Department of Child and Adolescent Psychiatry, Charité Universitätsmedizin Berlin, Berlin, Germany; ^5^Centre for Chronic Illness and Ageing, University of Greenwich, London, United Kingdom; ^6^Department of Brain and Behavioral Sciences, University of Pavia, Pavia, Italy; ^7^OASIS Service, South London and Maudsley NHS Foundation Trust, London, United Kingdom; ^8^National Institute for Health Research, Maudsley Biomedical Research Centre, London, United Kingdom

**Keywords:** antipyschotics, benzodiazepines (BDZ), first-episode, psychosis, treatment, electronic-health record (HER), first episode psychoses, cohort study

## Abstract

The impact of timing of antipsychotics and benzodiazepine treatment during a first episode of psychosis on clinical outcomes is unknown. We present a RECORD-compliant electronic health record cohort study including patients (*n* = 4,483, aged 14–35) with a primary diagnosis of any non-organic ICD-10 first episode of psychosis at SLAM-NHS between 2007 and 2017. The impact of antipsychotic timing (prescription > 1 week after a first episode of psychosis) was assessed on the primary outcome (risk of any psychiatric inpatient admission over 6 years), and secondary outcomes (cumulative duration of any psychiatric/medical/accident/emergency [A&E] admission over 6 years). The impact of prescribing benzodiazepine before antipsychotic at any point and of treatment patterns (antipsychotic alone, benzodiazepine alone, combination of antipsychotic with benzodiazepine) within the first week after a first episode of psychosis were also assessed. Survival analyses and zero-inflated negative binomial regressions, adjusted for core covariates, and complementary analyses were employed. Antipsychotic prescribed >1 week after a first episode of psychosis did not affect the risk of any psychiatric admission (HR = 1.04, 95% CI = 0.92–1.17, *p* = 0.557), but increased the duration of any psychiatric (22–28%), medical (78–35%) and A&E (30–34%) admission (months 12–72). Prescribing benzodiazepine before antipsychotic at any point did not affect the risk of any psychiatric admission (HR = 1.03, 95% CI = 0.94–1.13, *p* = 0.535), but reduced the duration of any psychiatric admission (17–24%, months 12–72), and increased the duration of medical (71–45%, months 12–72) and A&E (26–18%, months 12–36) admission. Prescribing antipsychotic combined with benzodiazepine within the first week after a first episode of psychosis showed better overall clinical outcomes than antipsychotic or benzodiazepine alone. Overall, delaying antipsychotic 1 week after a first episode of psychosis may worsen some clinical outcomes. Early benzodiazepine treatment can be considered with concomitant antipsychotic but not as standalone intervention.

## Introduction

Antipsychotic molecules are the mainstream pharma cological intervention for a First Episode of Psychosis (FEP) (1–8). Antipsychotics are highly effective for treating FEP, as quantified by recent large-scale meta-analyses (3, 9); the magnitude of antipsychotics’ efficacy is comparable to that of other drugs employed in somatic medicine (10). Although current clinical guidelines recommend oral antipsychotics as soon as a FEP is detected, they adopt a one-size-fits-all approach, largely tailored to the natural history of schizophrenia-spectrum psychoses, which tend to persist and lead life-time chronicity leading to high personal suffering (11), straining healthcare systems and society as a whole (12).

The current one-size-fits-all approach may be suboptimal as there is marked heterogeneity in the antipsychotic-specific effects between individuals with schizophrenia (13), supporting the growing consensus for the need to provide personalized treatments (14, 15). Moreover, current clinical guidelines are not prescriptive with respect to the specific timing of commencing antipsychotics during a FEP. In the absence of established biomarkers, diagnosis a FEP is entirely based on a careful psychopathological assessment that may lead to delays in initiating antipsychotics. For example, the FEP psychopathology is intrinsically heterogeneous extending from the typical positive psychotic symptoms to affective symptoms such as manic or depressive episodes (12, 16), which may require additional pharmacological treatments beyond antipsychotics. Indeed, about 66% of the incidence of clinical psychosis in the population is accounted for by preceding mood disorders (17–19). More on this, the duration of psychotic symptoms during a FEP may also be variable, with fleeting or spontaneous remission [about 19% of patients with a FEP (20)]. These cases are broadly classed as brief psychotic disorders (if drug-induced psychosis can be ruled out), variably operationalized as Acute and Transient Psychotic Disorders [ATPD in the ICD (21)], Brief Psychotic Disorders [BPD in the DSM (16)] and Brief (Limited) Intermittent Psychotic Disorders (BLIPS/BIPS) in the Clinical High Risk state for Psychosis (CHR-P) research paradigm [68% of BLIPS meet criteria for ATPD (22); for recent reviews of this topic see (20, 23–29)]. In other FEP cases, the duration of presenting psychotic symptoms may be transient and closely related to the pathophysiological effect of illicit drugs of abuse on the brain and vanish once the substance has been cleared from the body. Although brief psychotic FEP and drug-induced FEP can pose substantial risk of later developing schizophrenia [19 and 25%, respectively (29, 30)], there is no robust evidence that antipsychotics are effective in preventing these outcomes. Vice versa, these molecules are also associated to relevant side-effects, in particular in young people presenting with a FEP (31, 32).

A recent meta-analysis (33) showed that there is highly suggestive evidence for the relationship between a longer duration of untreated psychosis (DUP) in patients diagnosed with FEP, and more severe positive symptoms, more severe negative symptoms and lower chance of remission at follow-up (mean follow-up time: 6.8, 5.9, and 6.1 years, respectively). However, because of the dynamic course of the FEP presentation and challenging diagnosis, clinicians may be unwilling to immediately commence antipsychotic interventions upon detection of FEP cases and rather adopt watchful-waiting strategies. For example, in preventive CHR-P clinics, clinicians typically wait between 7 days (e.g., BLIPS) to 3 months (e.g., BIPS) before commencing an antipsychotic treatment; such timing pragmatically defines the cut-off point for transitioning from a state of CHR-P to FEP (24, 26, 34). In FEP patients hospitalized, similar watchful-waiting strategies may be adopted, in combination with benzodiazepines treatments to manage behavioral problems. There are no formal studies reporting on the prevalence of these prescription patterns in clinical practice. Benzodiazepine prescription may be effective in light of the emerging evidence on the role of GABAergic transmission in animal neurodevelopmental models of schizophrenia (35, 36). However, their clinical effectiveness as stand-alone treatment or in combination with antipsychotics around the FEP remains unknown (37). Therefore, the real-world timing and treatment pattern in FEP populations remains poorly understood to date.

In order to fill this gap of knowledge and due to the accumulating evidence regarding the long-term importance of early treatment decisions in FEP patients, the present study leverages data from Electronic Health Records (EHR) representing real-world clinical care in south-London to assess longitudinal outcomes over a 6-year follow-up period. The first (primary) aim was to test the impact of antipsychotic timing (prescription after the first week since FEP diagnosis, compared to within 1 week) on the risk of any (voluntary and compulsory) psychiatric inpatient admission over 6 years, as well as the secondary outcomes, while controlling for core confounders. We hypothesize that delaying antipsychotic prescription by 1 week from FEP diagnosis will be associated with an increased risk of psychiatric inpatient admission and worse secondary outcomes. The second aim was to test if there is an effect of prescribing benzodiazepines before (compared to after) antipsychotics at any point on these primary and secondary outcomes. The third aim was to test the impact of treatment patterns (combination of antipsychotics with benzodiazepine compared to antipsychotics alone or benzodiazepines alone) within the first week after FEP diagnosis on all the secondary outcomes.

## Materials and methods

This retrospective cohort study adhered to the Reporting of Studies Conducted Using Observational Routinely Collected Health Data statement (38) (Supplementary Table 1).

### Design and data source

We conducted a retrospective EHR cohort study (39), queried with the Clinical Record Interactive Search tool [CRIS (40)], extracting routine healthcare data and treatments records of patients accessing to the South London and Maudsley (SLaM) National Health Service (NHS) Foundation Trust (41). SLaM is a UK NHS mental health trust, providing secondary mental health care to a population around 1.36 million individuals in south London (Lambeth, Southwark, Lewisham, and Croydon boroughs), where the incidence of psychosis is particularly high (39). Details of the population size composing different boroughs within SLaM (39) and of local early intervention for psychosis pathways are available elsewhere (42, 43). SLaM is paper-free and all patients have a personal EHR, which clinicians continuously update throughout patients care, regardless of discharges from an/or referrals to other services. Since SLaM is the only secondary mental health service in this area, it includes both inpatients and outpatients.

All the ICD-10 diagnoses recorded in CRIS clinical care register are assigned by clinicians as part of their standard clinical practice, and no structured psychometric interviews were employed to ascertain the type of psychotic diagnoses. Therefore, while the psychotic diagnoses in our analyses are high in ecological validity (i.e., they represent real-world clinical practice), they have not been subjected to formal validation with research-based criteria (44). For example, clinician subjectivity, including structural or unconscious bias, can impact how diagnoses are recorded for given individuals thereby reducing standardization of output (45). However, the validity of FEP diagnoses has previously been considered acceptable to high within clinical care registers (46, 47). Notably, the use of structured diagnostic interviews can itself lead to selection biases, decreasing the transportability of models (48). There is also meta-analytical evidence indicating that within psychotic disorders, administrative data recorded in clinical registers are generally predictive of true validated diagnoses (49). To add further evidence, CRIS has also proven to be reliable for the diagnoses of other mental disorders (50).

Approval for the study was granted by the Oxfordshire Research Ethics Committee C; because the data set comprised deidentified data, informed consent was not required (40).

### Study population

All individuals receiving a diagnosis of FEP from 2007 to 2017 in SLaM and aged between 14 and 35 years [to represent the early intervention population (42, 43)] were included. The FEP diagnosis was operationalized as a primary diagnosis of any non-organic ICD-10 psychotic disorder: schizophrenia spectrum psychoses (F20.x, F25.x, except F20.4 and F20.5), acute and transient psychotic disorder (ATPD: F23.x), affective spectrum psychoses (F30.2, F31.2/5, F32.3, and F33.3), psychotic disorders due to psychoactive substance abuse (F10.5, F11.5, F12.5, F13.5, F14.5, F15.5, F16.5, and F19.5) and other psychotic disorders (F22.x, F24, F28, F29, and F53.1). Subjects were then excluded if they had been prescribed with antipsychotic treatment before 2007, as the CRIS system was set up only in 2007 and treatment regiments can only be considered to be reliable from that timepoint.

### Descriptive variables

Descriptive variables included: age, sex, ethnicity, employment, marital status, severity as measured by the Health of the Nation Outcome Scales (HONOS), type of antipsychotic molecule, type of FEP diagnosis [index ICD-10 (21) diagnostic code] and mean follow-up time. Full details of sociodemographic and clinical variables can be found in Tables 1A,B.

**TABLE 1:** Sociodemographic characteristics, clinical characteristics and exposure variables of study population (*n* = 4,483).

(A) Sociodemographic characteristics	

Characteristic	*N* = 4,483
**Age; mean (SD)**	25.2 (5.5)
**Gender [*n* (%)]**	
Male	2,659 (59.3)
Female	1,823 (40.7)
(Missing)	1 (0.0)
**Ethnicity [*n* (%)]**	
Caucasian	1,808 (40.3)
Black	1,711 (38.2)
Other/Mixed	545 (12.2)
Asian	333 (7.4)
(Missing)	86 (1.9)
**Employment [*n* (%)]**	
Other	898 (20.0)
Unemployed	403 (9.0)
Student	221 (4.9)
Employed	114 (2.5)
(Missing)	2,847 (63.5)
**Marital status [*n* (%)]**	
Single	3,621 (80.8)
In a relationship	389 (8.7)
Separated or divorced	127 (2.8)
(Missing)	346 (7.7)

**(B) Clinical characteristics**	

**HONOS (severity); mean (SD)**	11.4 (6.4)
(Missing)	648 (14.5)
**Type of first antipsychotic molecule [*n* (%)]**	
Olanzapine	1,944 (43.4)
Risperidone	1,070 (23.9)
Aripiprazole	589 (13.1)
Quetiapine	404 (9.0)
Haloperidol	203 (4.5)
Amisulpiride	140 (3.1)
Zuclopenthixol	32 (0.7)
Chlorpromazine	24 (0.5)
Flupenthixol	23 (0.5)
Paliperidone	14 (0.3)
Pipotiazine	10 (0.2)
Trifluoperazine	9 (0.2)
Prochlorperazine	5 (0.1)
Sulpiride	5 (0.1)
Fluphenazine	4 (0.1)
Perphenazine	2 (0.0)
Ziprasidone	2 (0.0)
Levomepromazine	1 (0.0)
Novorapid	1 (0.0)
NovoRapid FlexPen solution for injection	1 (0.0)
**FEP diagnosis cluster [*n* (%)]**	
Schizophrenia	1,729 (38.6)
Other psychotic disorders	1,047 (23.4)
Acute and transient psychosis	875 (19.5)
Affective psychosis	614 (13.7)
Substance induced psychosis	218 (4.9)
**Follow-up time (weeks); mean (SD)**	168.9 (145.6)

**(C) Exposure variables**	

**Antipsychotic timing; mean (SD)**	
AP within 1 week from FEP diagnosis	3,908.0 (87.2)
AP more than 1 week from FEP diagnosis	575.0 (12.8)
**Antipsychotic before (or after) benzodiazepine at any point; mean (SD)**	
AP before BDZ	2,944.0 (65.7)
BDZ before AP	1,539.0 (34.3)
**Treatment pattern within first week after FEP diagnosis; mean (SD)**	
AP and BDZ	3,181.0 (71.0)
AP alone	727.0 (16.2)
none	390.0 (8.7)
BDZ alone	185.0 (4.1)

Continuous variables are described as mean (SD). Categorical variables (and missing data) are described as *n* (%). HONOS, Health of the Nation Outcome Scales; AP, antipsychotic; BDZ, benzodiazepine.

### Exposure variables

To define the exposure variable in the primary aim (impact of antipsychotic timing), we extracted the time to first antipsychotic from an index FEP diagnosis, and dichotomized it at 1 week (≤1 or >1 week). This decision was done to reflect clinical practice in CHR-P clinics where the psychosis threshold is <1 week for BLIPS (22). Similarly SLaM clinicians may adopt watchful waiting periods of less than 1 week for FEP patients admitted to hospitals. For the second aim, we determined whether benzodiazepines were prescribed before antipsychotics (or vice versa) by extracting the date of first benzodiazepine prescription. For the third aim, treatment pattern within the first week from diagnosis was computed as follows: benzodiazepine only, antipsychotic only or a combination of both benzodiazepine and antipsychotic. Full details of the exposure variables can be found in Table 1C.

### Primary and secondary outcomes

The primary outcome was the risk of any (voluntary and compulsory) psychiatric inpatient admission over 6 years after FEP diagnosis. The secondary outcomes were defined as the cumulative duration (in days) of any (voluntary and compulsory) psychiatric, medical (non-mental health), and accident/emergency (A&E) admission every 12 months up to 72 months after FEP diagnosis.

### Statistical analysis

The first aim was to assess the impact of antipsychotic timing on all the clinical outcomes outlined above. To assess its impact on the primary outcome, we described the cumulative probability of risk of any psychiatric inpatient admission over 6-year for patients given antipsychotics within ≤1 or >1 week after FEP diagnosis, using Kaplan-Meier survival functions (51) and Greenwood 95% confidence intervals (52). After checking the proportional hazards assumption, we used Cox proportional hazards multivariate regressions to evaluate the effect of antipsychotic timing (≤1 vs >1 week) on the probability of any (voluntary or compulsory) inpatient psychiatric admission (event) and the time to admission from the FEP diagnosis, adjusting for well-known confounders: age, sex, type of ICD-10 diagnosis, and severity of presentation (closest available HONOS score to FEP diagnosis date), as these variables have been significantly associated with clinical outcomes in FEP. Specifically, being male (53, 54), receiving a diagnosis of non-affective psychosis (55) and showing more severe baseline HONOS scores (53, 56, 57) have been associated with worse clinical outcomes, whereas age at baseline has shown mixed effects (56, 58). To assess the impact of antipsychotic timing on secondary outcomes, a zero-inflated negative binomial regression model (adjusted with the same core covariates) was adopted due to the excess of zero values (reflecting a subset of participants without any admissions) and overdispersion (59). The same analyses were repeated to test the impact of prescribing benzodiazepines before antipsychotics at any point (aim 2) and of treatment patterns within the first week after FEP (aim 3) on all clinical outcomes.

### Sensitivity analyses

Finally, three sensitivity analyses were conducted. The first sensitivity analysis was run to examine the effect of antipsychotic timing (aim 1), prescribing benzodiazepines before antipsychotics at any point (aim 2) and treatment patterns within the first week after FEP (aim 3) on the risk and duration of psychiatric admission stratified by compulsory (only) or voluntary (only) admission. The second sensitivity analysis was conducted to examine the effect of antipsychotic timing (aim 1), prescribing benzodiazepines before antipsychotics at any point (aim 2) and treatment patterns within the first week after FEP (aim 3) on the risk of any psychiatric admission at 1, 2, 3, 4, and 5 years after FEP diagnosis. The aim of this sensitivity analysis was to explore whether the exposure variables have an effect on the primary outcome in the shorter term. The third sensitivity analysis was conducted to test the effect of antipsychotic timing (aim 1), prescribing benzodiazepines before antipsychotics at any point (aim 2) and treatment patterns within the first week after FEP (aim 3) on the secondary outcomes within the sub-group of subjects with a diagnosis of an Acute and Transient Psychotic Disorder (ATPD), to explore how decisions regarding timing of treatment may specifically impact brief episodes of psychoses that share similarities with BLIPS/BIPS (29).

### Supplementary analyses

The demographic characteristics (age, gender, marital status, ethnicity, and employment) and clinical characteristics (follow-up time, severity [HONOS], antipsychotic or benzodiazepine as first treatment, type of first antipsychotic molecule, and FEP diagnosis) were compared between individuals who received antipsychotics within the first week and after 1 week from FEP diagnosis using a one-way ANOVA test for continuous variables and chi-squared tests for categorical variables. Similarly, individuals who were prescribed with benzodiazepine before antipsychotics (at any point) compared to those who were prescribed with antipsychotics before benzodiazepines (at any point) were compared.

For all analyses, two-sided *P*-values less than 0.01 were considered significant. All analyses were conducted in R (60).

## Results

### Sample characteristics

**FIGURE 1:**
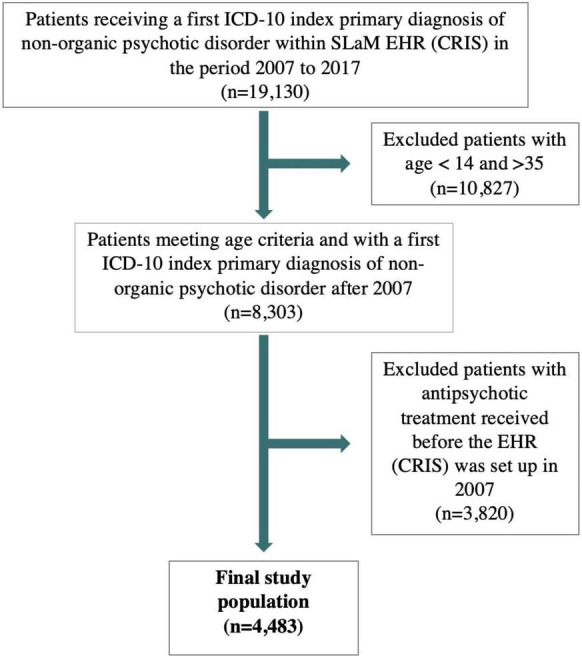
Flow-chart of study population. ICD, internal classification of diseases; SLaM, South London and Maudsley.

Out of 19,130 subjects receiving a FEP diagnosis, 10,827 were excluded because they were younger than 14 or older than 35 years old, 3,820 because they received antipsychotic treatment before 2007 (Figure 1). The final database included 4,483 FEP subjects with a mean age of 25.3 years (SD = 5.5) and 59.5% were males. Most subjects were of non-Caucasian ethnicity (58.3%), of unknown employment (55%) and single (80.6%). Across the FEP cases, 38.6% were schizophrenia or schizoaffective disorders, 19.5% ATPD, 13.7% mood disorders with psychotic features, 4.9% psychotic disorders due to psychoactive substance abuse, and 23.3% had other types of psychotic disorders (detailed ICD-10 diagnostic distributions are listed in Supplementary Table 2).

In terms of medication, antipsychotics were prescribed within 1 week from diagnosis in 87.2% of the sample, with a minority of patients (12.8%) receiving prescription of antipsychotics >1 week after diagnosis of psychosis. Approximately two thirds of the sample (65.7%) received antipsychotics before benzodiazepines, whilst one third (34.3%) were prescribed with benzodiazepines first. The most frequently prescribed antipsychotic molecule during a FEP was olanzapine (42.5%), followed by risperidone (23.4%) and aripiprazole (12.9%). Regarding the pattern of treatment prescription within the first week after FEP, most subjects received a combination of antipsychotics and benzodiazepines (71.0%), whereas 16.2% received only antipsychotics, 4.1% received only benzodiazepines and 8.7% received no treatment prescription. The cohort was followed up for a mean of 168.9 weeks (SD:145.6). Full details of socio-demographic and clinical characteristics of the included sample are reported in Table 1.

In the supplementary analyses, some differences emerged in demographic (i.e., age, marital status) and clinical characteristics (i.e., severity, first treatment [antipsychotics vs benzodiazepines], type of first antipsychotic molecule, diagnosis and follow-up time) between subjects prescribed with antipsychotics within versus after 1 week from FEP diagnosis (Supplementary Datasheet 1). However, no differences emerged in demographic characteristics between subjects prescribed with antipsychotics before benzodiazepines at any point compared to subjects prescribed with benzodiazepines before antipsychotics at any point (Supplementary Datasheet 2), but there were some clinical differences (i.e., delay to antipsychotic commencement, type of first antipsychotic molecule, FEP diagnosis cluster and follow-up time).

#### Effect of antipsychotic timing on clinical outcomes

Regarding the primary outcome, prescribing antipsychotics more than 1 week after diagnosis was not associated with an increased risk of any inpatient admission compared to within 1 week (HR = 1.04, 95% CI = 1.92–1.17, *p* = 0.557) Cox regression (Table 2A). For the covariates, Table 2A shows that older age (HR = 0.99, 95% CI = 0.98–1.00, *p* = 0.002) is linked to lower risk of any admission, whereas severity (HR = 1.01, 95% CI = 1.01–1.02, *p* < 0.001) and being male (HR = 1.14, 95% CI = 1.04–1.25, *p* = 0.004) were linked to higher risk of any admission. No significant associations were found for type of ICD FEP diagnosis.

**TABLE 2:** Adjusted multivariable Cox regression results. The primary outcome (risk of any psychiatric inpatient admission over 6 years after FEP diagnosis) was tested for the effect of **(A)** antipsychotic timing (subjects = 3,834, 2,128 admissions), **(B)** prescribing benzodiazepine before antipsychotics (subjects = 3,834, admissions = 2,128), and **(C)** treatment patterns within the first week after FEP (*n* = 3,512, admissions = 1,887).

(A)				

Factor		HR	95% CI	*P*-value
Antipsychotic more than 1 week after diagnosis (vs ≤ 1 week)		1.04	0.92–1.17	0.557
Male sex (vs female)		1.14	1.04–1.25	**0.004**
Age (continuous)		0.99	0.98–1.00	**0.002**
ICD diagnosis (vs ATPD)	Affective psychosis	0.83	0.71–0.97	0.020
	Other psychotic disorders	0.95	0.83–1.08	0.426
	Schizophrenia	0.97	0.86–1.09	0.603
	Substance-induced psychosis	1.04	0.85–1.28	0.695
Severity (HONOS)		1.01	1.01–1.02	**<0.001**

**(B)**				

Prescribing benzodiazepine before (vs after) antipsychotics (at any point)		1.03	0.94–1.13	0.535
Male sex (vs female)		1.14	1.04–1.25	**0.004**
Age (continuous)		0.99	0.98–1.00	**0.001**
ICD diagnosis (vs ATPD)	Affective psychosis	0.83	0.71–0.97	0.020
	Other psychotic disorders	0.95	0.84–1.08	0.449
	Schizophrenia	0.97	0.87–1.09	0.667
	Substance-induced psychosis	1.04	0.85–1.29	0.680
Severity (HONOS)		1.01	1.01–1.02	**<0.001**

**(C)**				

Combination of antipsychotics with benzodiazepine treatment (vs antipsychotics alone)		1.05	0.94–1.17	0.403
Combination of antipsychotics with benzodiazepine treatment (vs benzodiazepine alone)		1.07	0.86–1.32	0.538
Male sex (vs female)		1.14	1.04–1.25	**0.007**
Age (continuous)		0.98	0.98–0.99	**0.003**
ICD diagnosis (vs ATPD)	Affective psychosis	0.83	0.71–0.98	0.030
	Other psychotic disorders	0.93	0.81–1.06	0.267
	Schizophrenia	0.95	0.84–1.08	0.428
	Substance-induced psychosis	0.97	0.78–1.22	0.821
Severity (HONOS)		1.01	1.01–1.02	**<0.001**

Statistically significant results (*p* < 0.01) are shown in bold. ATPD, acute and transient psychotic disorder; CI, confidence interval; HONOS, Health Of the Nation Outcome Scales; ICD, Internal Classification of Diseases; HR, hazard ratio.

**FIGURE 2:**
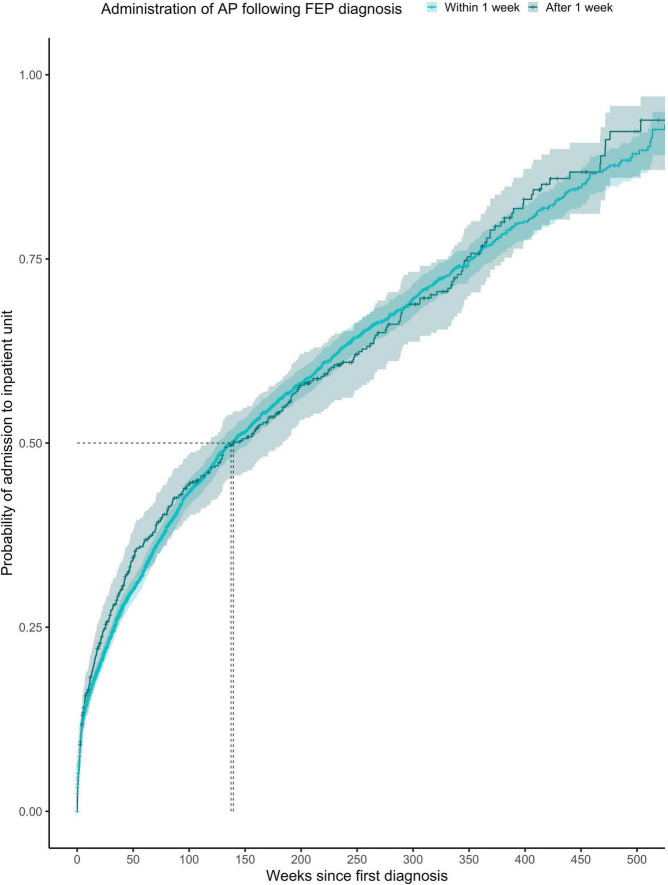
Kaplan-Meier survival curve showing cumulative probability of any psychiatric admission over time (weeks) after FEP diagnosis for patients given antipsychotics within ≤1 week or >1 week after diagnosis. Greenwood 95% confidence intervals are displayed for each curve. The dashed lines indicate the median time to admission in each group.

The proportionality assumption was met (χ^2^ = 0.09, *p* = 0.77), suggesting that the hazard ratios were likely to be constant over time (Figure 2). The median time to any admission was 137 weeks (95% CI = 126–153) for subjects prescribed with antipsychotics within the first week from FEP diagnosis (1,945 admitted out of 3,908 subjects) and 139 weeks (95% CI = 116–184) for subjects prescribed with antipsychotics after the first week from FEP diagnosis (369 admitted out of 575 subjects).

Regarding the secondary outcomes (Table 3), the zero inflated negative binomial regression adjusted for age, sex, severity and diagnosis, showed that prescribing antipsychotics >1 week compared within 1 week was significantly associated with 22, 35, 28, 33, 26, and 28% more days of any psychiatric admission at a follow-up of 12, 24, 36, 48, 60, and 72 months, respectively, with 78, 77, 62, 66, 54, and 35% more days of medical admission (12, 24, 36, 48, 60, and 72 months), 30, 30, 33, and 34% more days in A&E (36, 48, 60, and 72 months).

**TABLE 3:** Zero-inflation negative binomial regressions to assess the effect of exposure variables (antipsychotic timing, prescribing benzodiazepine before antipsychotics at any point and treatment patterns within first week after diagnosis) on secondary outcomes, adjusted for age, sex, severity, and diagnosis.

		Aim 1				Aim 2				Aim 3							
				
		Antipsychotic timing (>1 week vs < 1 week after diagnosis) (*N* = 3,834)				Prescribing benzodiazepine before antipsychotics (at any point) (*N* = 3,834)				Treatment pattern within first week after diagnosis (AP + BDZ vs AP alone) (*N* = 3,512)				Treatment pattern within first week after diagnosis (AP + BDZ vs BDZ alone) (*N* = 3,512)			
					
Duration of admission (days)	months	IRR	CI-L	CI-H	*P*-value	IRR	CI-L	CI-H	*P*-value	IRR	CI-L	CI-H	*P*-value	IRR	CI-L	CI-H	*P*-value
Any psychiatric admission	12	1.22	1.06	1.4	**0.005**	0.83	0.76	0.91	**<0.001**	0.77	0.68	0.88	**<0.001**	1.05	0.84	1.32	0.647
	24	1.35	1.17	1.56	**<0.001**	0.77	0.69	0.84	**<0.001**	0.78	0.68	0.88	**<0.001**	1.07	0.84	1.35	0.59
	36	1.28	1.11	1.47	**0.001**	0.75	0.68	0.83	**<0.001**	0.82	0.72	0.93	**0.002**	1.35	0.98	1.58	0.072
	48	1.33	1.15	1.54	**<0.001**	0.74	0.67	0.81	**<0.001**	0.81	0.72	0.92	**0.001**	1.22	0.96	1.54	0.107
	60	1.26	1.09	1.45	**0.002**	0.75	0.68	0.83	**<0.001**	0.83	0.73	0.95	**0.007**	1.31	1.02	1.67	0.033
	72	1.28	1.1	1.48	**0.001**	0.76	0.68	0.84	**<0.001**	0.86	9,75	0.98	0.022	1.35	1.05	1.73	0.021
Medical non-MH admission	12	1.78	1.64	1.93	**<0.001**	1.71	1.6	1.82	**<0.001**	1.13	0.85	1.49	0.405	0.56	0.24	1.34	0.194
	24	1.77	1.66	1.89	**<0.001**	1.53	1.45	1.61	**<0.001**	1.15	0.92	1.45	0.226	0.68	0.34	1.35	0.269
	36	1.62	1.54	1.71	**<0.001**	1.41	1.35	1.47	**<0.001**	**1.33**	**1.09**	**1.63**	**0.005**	0.76	0.43	1.34	0.336
	48	1.66	1.57	1.76	**<0.001**	1.41	1.35	1.48	**<0.001**	1.26	1.02	1.56	0.032	82	0.44	1.52	0.53
	60	1.54	1.46	1.62	**<0.001**	1.41	1.35	1.47	**<0.001**	**1.37**	**1.12**	**1.66**	**0.002**	0.67	0.39	1.17	0.157
	72	1.35	1.29	1.42	**<0.001**	1.45	1.4	1.51	**0.001**	**1.4**	**1.15**	**1.7**	**<0.001**	0.74	0.43	1.28	0.288
A&E admission	12	1.23	1.02	1.48	0.031	1.26	1.11	1.43	**<0.001**	1.24	1.04	1.48	0.014	0.6	0.44	0.81	**<0.001**
	24	1.23	1.04	1.45	0.013	1.25	1.12	1.41	**<0.001**	1.2	1.03	1.39	0.018	0.66	0.51	0.87	**0.003**
	36	1.3	1.12	1.52	**<0.001**	1.18	1.06	1.31	**0.003**	1.13	0.98	1.3	0.09	0.69	0.53	0.9	**0.006**
	48	1.3	1.12	1.51	**<0.001**	1.11	1	1.23	0.055	1.04	0.91	1.19	0.579	0.74	0.58	0.95	0.017
	60	1.33	1.14	1.54	**<0.001**	1.09	0.98	1.21	0.113	1.03	0.9	1.17	0.704	0.72	0.56	0.93	0.011
	72	1.34	1.15	1.55	**<0.001**	1.07	0.97	1.19	0.176	0.97	0.85	1.11	0.681	0.72	0.56	0.92	0.01

Reference group; IRR < 1 indicates more favourable effects (fewer days) for AP > 1 week (aim 1), BDZ given first (aim 2) or AP + BDZ (aim 3). Statistically significant results (*p* < 0.01) are shown in bold. IRR, incidence rate ratio; CI-L, 95% confidence interval (lower); CI-H, 95% confidence interval (higher); AP, antipsychotic; BDZ, benzodiazepine; non-MH, non-mental health; A&E, accident and emergency.

#### Effect of prescribing benzodiazepines before antipsychotics on clinical outcomes

Regarding the primary outcome, prescribing benzodiazepines before (vs after) antipsychotics at any point was not significantly associated with an increased risk of any inpatient admission in the adjusted (HR = 1.03, 95% CI = 0.94–1.13, *p* = 0.535) Cox regression models (Table 2B). Similar effects from the covariates emerged: older age (HR = 0.99, 95% CI = 0.98–1.00, *p* = 0.001) is linked to lower risk of any admission, whereas severity (HR = 1.01, 95% CI = 1.01–1.02, *p* < 0.001) and being male (HR = 1.14, 95% CI = 1.04–1.25, *p* = 0.004) were linked to higher risk of any admission. No significant associations were found for type of ICD FEP diagnosis.

**FIGURE 3:**
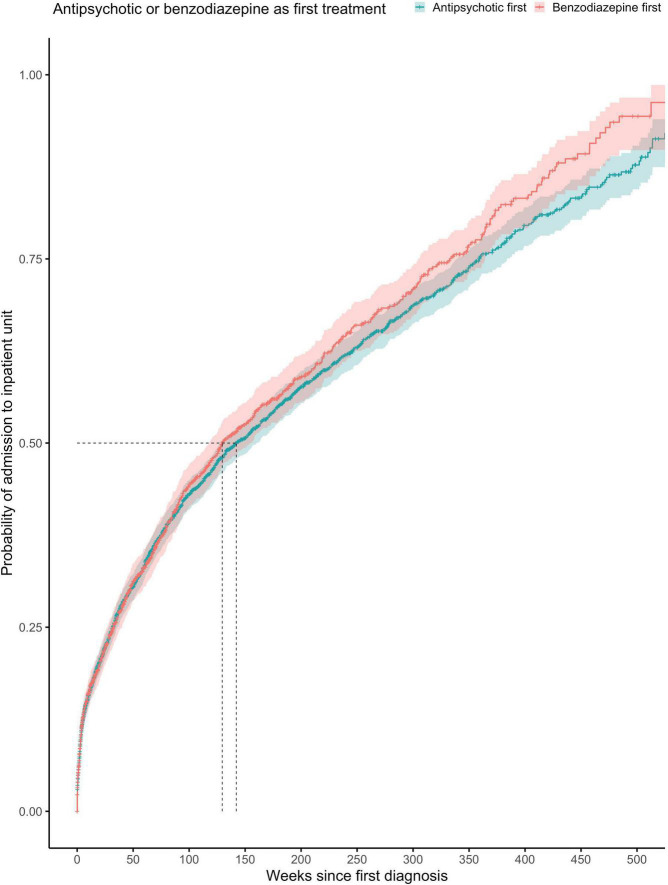
Kaplan-Meier survival curve showing cumulative probability of any psychiatric admission over time (weeks) after FEP diagnosis for patients prescribed with antipsychotics or benzodiazepine as first treatment (at any point). Greenwood 95% confidence intervals are displayed for each curve. The dashed lines indicate the median time to admission in each group.

The proportionality assumption was met (χ^2^ = 4.86, *p* = 0.027), suggesting that these hazard ratios were likely to be constant over time (Figure 3). The median time to any admission was 142 weeks (95% CI = 129–160) for subjects prescribed with antipsychotics with antipsychotics before benzodiazepines at any point (1,529 admitted out of 2,944 subjects) and 130 weeks (95% CI = 118–154) for subjects prescribed with benzodiazepines before antipsychotics at any point (785 admitted out of 1,539 subjects).

Regarding the secondary outcomes (Table 3), the zero inflated negative binomial regression adjusted for age, sex, severity and diagnosis, showed that prescribing benzodiazepines before antipsychotics at any point was significantly associated with 17, 23, 25, 26, 25, and 24% fewer days of any psychiatric admission, but with 71, 53, 41, 41, 41, and 45% more days of medical admission (12, 24, 36, 48, 60, and 72 months), and 26, 25, and 18% more days in A&E (12, 24, and 36 months).

#### Effect of treatment pattern within first week after first episode of psychosis diagnosis on clinical outcomes

Regarding the primary outcome, prescribing antipsychotics in combination with benzodiazepines compared to antipsychotics alone within the first week after diagnosis was not associated with a reduced risk of any psychiatric admission in the adjusted (HR = 1.05, 95% CI = 0.94–1.17, *p* = 0.403) Cox regression (Table 2C). Similarly, prescribing antipsychotics in combination with benzodiazepines compared to benzodiazepine alone did not show a significant association to the risk of any psychiatric admission (HR = 1.07, 95% CI = 0.86–1.32, *p* = 0.538). For the covariates in the adjusted model, Table 2C shows that similar effects from the covariates emerged: older age (HR = 0.98, 95% CI = 0.98–0.99, *p* = 0.003) is linked to lower risk of any admission, whereas severity (HR = 1.01, 95% CI = 1.01–1.02, *p* < 0.001) and being male (HR = 1.14, 95% CI = 1.04–1.25, *p* = 0.007) were linked to higher risk of any admission. No significant associations were found for type of ICD FEP diagnosis.

**FIGURE 4:**
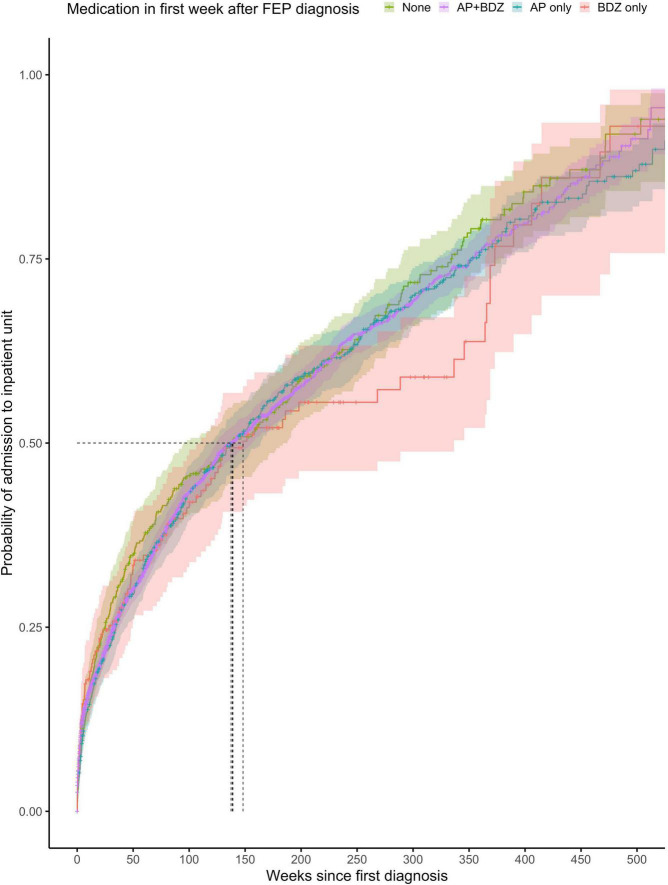
Kaplan-Meier survival curve showing cumulative probability of any psychiatric admission over time (weeks) after FEP diagnosis for patients prescribed with no medication, combination of antipsychotics and benzodiazepines or antipsychotics alone or benzodiazepines alone within the first week after FEP diagnosis. Greenwood 95% confidence intervals are displayed for each curve. The dashed lines indicate the median time to admission in each group.

The proportionality assumption was met (χ^2^ = 4.84, *p* = 0.92), suggesting that the hazard ratios were likely to be constant over time (Figure 4). The median time to any admission was 139 weeks (95% CI = 95–188) for subjects prescribed with no treatment within the first week from diagnosis (268 admitted out of 390 subjects), 137 weeks (95% CI = 125–156) for subjects prescribed with a combination of antipsychotics and benzodiazepines within the first week from diagnosis (1,463 admitted out of 3,181 subjects), 139 weeks (95% CI = 118–164) for subjects prescribed with antipsychotics only within the first week from diagnosis (482 admitted out of 727 subjects), and 148 weeks (95% CI = 111–337) for subjects prescribed with benzodiazepines only within the first week from FEP diagnosis (101 admitted out of 185 subjects).

Regarding the secondary outcomes, the zero inflated negative binomial regression adjusted for age, sex, severity and diagnosis, showed that prescribing a combination of benzodiazepines and antipsychotics compared to antipsychotics alone within the first week was significantly associated with 23, 22, 18, 19, and 17% fewer days of any psychiatric admission at a follow-up of 12, 24, 36, 48, and 60 months, respectively, but 33, 37, and 40% more days of medical admission at 36, 60, and 72 months, and no significant effects on the duration of A&E duration of admission (Table 3). In contrast, prescribing a combination of benzodiazepines and antipsychotics compared to antipsychotics alone was associated with shorter duration of A&E admission (40, 34, and 31% at 12, 24, and 36 months after FEP diagnosis), but no significant effects on the duration in psychiatric or medical admission.

### Sensitivity analyses A: Stratification of any psychiatric admission by compulsory (only) or voluntary (only) admission

For aim 1, prescribing antipsychotics more than 1 week after diagnosis did increase the risk of voluntary admission (HR = 1.27, 95% CI = 1.12–1.44, *p* < 0.001) but not compulsory admission (HR = 0.94, 95% CI = 0.82–1.08, *p* = 0.380; Supplementary Table 3), and continued to be significantly associated with increased days in admission for both voluntary (48–45% [months 12–72]) and compulsory (35–28% [months 24–60]) admissions (Supplementary Table 6).

For aim 2, prescribing benzodiazepines before (vs after) antipsychotics at any point did not affect the risk of voluntary admission (HR = 1.00, 95% CI = 0.90–1.10, *p* = 0.920) or compulsory admission (HR = 0.94, 95% CI = 0.82–1.08, *p* = 0.380; Supplementary Table 4), and continued to be significantly associated with fewer days in admission for both voluntary (21–31% [months 24–72]) and compulsory (17–17% [months 12–72]) admissions (Supplementary Table 6).

For aim 3, prescribing antipsychotics in combination with benzodiazepines was not associated with a reduced risk of voluntary (only) (HR = 0.87, 95% CI = 0.78–0.19, *p* = 0.019) or compulsory (only) (HR = 0.99, 95% CI = 0.88–1.12, *p* = 0.953) psychiatric admission (Supplementary Table 5). A combination of benzodiazepines and antipsychotics compared to AP alone within the first week continued to be significantly associated with shorter days in both voluntary psychiatric admission (by 24, 22, and 20% [months 12, 24 and 36]) and compulsory psychiatric admission (by 26 and 23% [months 12 and 24]). Similarly, to the primary analysis, a combination of benzodiazepines and antipsychotics compared to BDZ alone was not significantly associated with the duration of either voluntary or compulsory psychiatric admission (Supplementary Table 6).

### Sensitivity analyses B: Stratification of primary outcome by year after first episode of psychosis diagnosis

When we explored the effect of (1) antipsychotic timing, (2) prescribing benzodiazepine before antipsychotics, and (3) treatment patterns within the first week after FEP, on the risk of any psychiatric admission at 1, 2, 3, 4, and 5 years of follow-up, the lack of significant associations observed at 6-years were largely replicated, with the following exceptions observed at shorter follow-up times.

Prescribing antipsychotics more than 1 week after diagnosis (vs within 1 week) was associated with an increased risk in any inpatient admission after 2 years (HR = 1.29, 95% CI = 1.11–1.49, *p* < 0.001) and 3 years (HR = 1.31, 95% CI = 1.14–1.51, *p* < 0.001) from FEP diagnosis (Supplementary Table 7A), but continued to be insignificant at 1, 4, and 5 years. Prescribing benzodiazepines before (vs after) antipsychotics at any point was not significantly associated with risk of any inpatient admission at 1, 2, 3, 4, and 5 years from FEP diagnosis (Supplementary Table 7B). Prescribing combinatorial treatment within the first week was associated with a reduced risk of any inpatient admission at 1 year (HR = 0.76, 95% CI = 0.65–0.89, *p* < 0.001), 2 years (HR = 0.80, 95% CI = 0.70–0.92, *p* = 0.001) and 3 years (HR = 0.83, 95% CI = 0.73–0.94, *p* = 0.004) from FEP diagnosis when compared to antipsychotics alone, but not at 4 and 5 years (Supplementary Table 7C). Prescribing combinatorial treatment compared to benzodiazepines alone within the first week was not significantly associated with the risk of any inpatient admission at 1, 2, 3, 4, or 5 years after FEP diagnosis (Supplementary Table 7C). Overall these findings confirm the main analyses focused on 6 years follow-up.

### Sensitivity analyses C: Subset of subjects with acute and transient psychotic disorders diagnosis

When repeating the analyses in the subgroup of patients who had received an F23.x diagnosis (*n* = 783; Supplementary Table 8), the only associations that were replicated in magnitude and significance was the shorter duration of medical admission at 24 months (by 137%) after FEP diagnosis, when prescribed with benzodiazepines before antipsychotics (at any point), as well as the longer duration of medical admission (by 186, 116, and 77% at 12, 24, and 48 months) associated with prescribing antipsychotics with benzodiazepines (compared to antipsychotics alone). No significant associations were sustained, however, for other outcomes.

## Discussion

To the best our knowledge, this is the first study leveraging real world clinical data to compare the effect of timing and different patterns of antipsychotic and benzodiazepine prescription following a first diagnosis of psychosis on a range of clinical outcomes over time.

Firstly, we showed that prescribing antipsychotic treatment more than 1 week after a FEP diagnosis (compared to within the first week) did not affect the risk of any psychiatric admission over the next 6 years. However, delaying antipsychotic treatment was associated with an increase in the duration of any psychiatric (22–28%), medical (35–78%) and/or A&E (30–34%) admission each year over the following 6 years after FEP diagnosis. We believe these results are clinically relevant for the following reasons. To begin with, current guidelines leave the choice on timing of antipsychotics prescription entirely to clinicians’ judgments, where an antipsychotic-free observation period is recommended (61). However, we show that while starting medication more than 1 week after diagnosis did not affect the risk of psychiatric admission in the long term, such delay was associated with an increase in the risk of inpatient admission in the shorter term (i.e., at 2 and 3 years follow-up), and additionally was associated with an increase in the duration of any psychiatric, medical and A&E admissions in the short and long-term compared to prescribing the treatment within the first week following FEP diagnosis. This finding is relevant to the field, as previous studies have shown shorter duration of untreated psychosis to be linked to improved treatment response (62), but no guidelines delve into timing of treatment prescription with this granularity. Furthermore, as evidenced by our sensitivity analyses, antipsychotic prescription 1 week after a FEP diagnosis exerted a differential effect on the type of psychiatric admission at 6-years of follow-up: it had no effect on the risk of compulsory admission but was associated with an increase in the risk of voluntary admission and durations of both compulsory and voluntary psychiatric admissions.

Secondly, prescribing benzodiazepines before (compared to after) antipsychotics at any point did not have an effect on the risk of any psychiatric admission but did show mixed effects on the durations of admission in different clinical settings (i.e., was significantly associated with shortened duration of psychiatric admission but extended medical and A&E admissions). We show formal evidence for the prescription pattern in FEP, as approximately two thirds of the sample (65.7%) received antipsychotics before benzodiazepines, whilst one third (34.3%) were prescribed with benzodiazepines first. To our best knowledge, it is the first time that empirical research demonstrates that in about one third of the first episode cases, the first treatment is a benzodiazepine rather than an antipsychotic. We further show that if benzodiazepines are prescribed before (vs after) antipsychotics at any point, they do not affect the risk of any psychiatric admission over 6 years after FEP diagnosis. Rather, if benzodiazepines are taken before antipsychotics at any point, the duration of psychiatric admission is reduced. These findings were observed for both compulsory and voluntary psychiatric admissions when analyzed separately in the sensitivity analysis. Although it is difficult to make causal inference in the context of a naturalistic study where multiple potential confounders remain unexplored, this finding aligns with the potential role of benzodiazepines in preventing the development of neuroanatomical and neurophysiological abnormalities associated with schizophrenia, as shown in animal models (63, 64). Benzodiazepines may exert this protective role by acting as GABA_*A*_ receptor agonists (65). However, a recent meta-analysis based on *in vivo* GABA neuroimaging studies did not show evidence for significantly altered GABA+ concentrations or GABA_*A*_ receptor availability in patients with schizophrenia compared to healthy volunteers (66). Therefore, although our results may align with a potential clinical effect of benzodiazepines on psychotic outcomes, the exact nature of this effect and the underlying neurobiological mechanism remain unknown. Additionally, benzodiazepine prescription before antipsychotics at any point was associated with a prolonged duration of medical and A&E admission, potentially due to their adverse side effects (33).

Thirdly, prescribing antipsychotics with benzodiazepines within the first week after FEP diagnosis compared to monotherapy treatment (antipsychotics alone or benzodiazepines alone), was not associated with a reduced risk of any psychiatric admission over 6 years after FEP diagnosis. However, in the shorter term, combination therapy compared to antipsychotics alone was associated with a reduced risk of inpatient admission (1–3 years), as well as the duration of psychiatric admission (1–5 years), but increased the duration of medical admission (over 3,5, and 6 years after FEP). In contrast, combination therapy compared to benzodiazepines alone reduced the duration of A&E admission only. One possible interpretation is that these treatment patterns may reflect confounding by indication whereby patients with somatic conditions or behaviorally agitated may be more likely to be prescribed benzodiazepine. Given this uncertainty, caution is required and benzodiazepines should not be encouraged as stand-alone treatment for FEP due to their potential detrimental effects on the duration of A&E and medical admissions. Vice versa, if taken in combination with antipsychotics within the first week, the clinical outcomes appear better. Combination treatment within the first week was associated with overall reductions in all secondary outcomes relative to no treatment and reduced the duration of psychiatric admission relative to antipsychotics alone at the cost of prolonging the duration of medical admission.

Interestingly, when focusing only on subjects with an ATPD diagnosis in the sensitivity analysis, the beneficial effects of early treatment disappear; prescribing antipsychotics within the first week from FEP diagnosis, benzodiazepine treatment before antipsychotics at any point and combinatorial treatment within the first week are no longer associated with shorter durations of psychiatric, medical or A&E admissions. This finding, in conjunction with the risk of exposing approximately half of the ATPD population to unnecessary antipsychotic treatment [due to their variable likelihood of developing a relapse (20, 22, 26, 29, 67, 68)], confirms that early antipsychotic prescription in brief psychotic episodes that have remitted within a relatively short period of time may not be vital to optimize long-term disease outcomes. Some evidence suggests that excessive prescription of antipsychotics may actually increase the risk of developing persistent psychotic disorders (such as schizophrenia) in ATPD patients (69).

### Limitations

This work has several limitations. First, our naturalistic design is high in ecological validity but has some inherent limitations, including several unaddressed confounding factors (e.g., substance use and symptomatology) which could not be tested in the current dataset. We controlled our multivariable models for the most widely known factors impacting clinical outcomes in this population. Further research could refine these analyses using Natural Language Processing (NLP) techniques (70) to longitudinally compare the symptomatology trajectories of subjects with different timing and pattern of medication [similarly NLP algorithms have enhanced the prognostic accuracy of a psychosis risk calculator by extract symptom data from EHRs (45)]. Moreover, although we have focused our research question on the effects of early treatment prescription on long-term outcomes, it is certainly possible that other factors in particular relating to subsequent treatments received over the follow-up time may have driven some of the longitudinal outcomes of interest. For example, side effects associated with medications or improvement of symptoms may have led clinicians to change the patient’s prescription over time. There are also several other unmeasurable factors (e.g., treatment compliance by the patients or engagement with SLaM mental health services) that could also have had an effect on the observed outcomes. As we have explicitly acknowledged, the naturalistic design of the current study does not allow formulating causal inference, and our findings are limited to association-level. Second, the severity of presentation can drive prescription timing and patterns, introducing confounding by indication, as we acknowledged above. We accounted for ecologically collected functioning measure (HONOS), which should at least moderate the confounding role of symptoms severity, as functioning is closely correlated with symptoms (71, 72). Third, in everyday clinical practice, registering a subject’s diagnosis might be delayed due to clinical/administrative reasons. Hence it is possible that some of the “first” diagnoses of psychoses included in this work might have indeed had previous psychotic episodes. To account for this we restricted our inclusion to subjects with age between 14 and 35, which represents the epidemiological peak age range for the onset of psychotic disorders (73, 74). Overall, our findings should be replicated in external samples, to ensure cross-regional/national validity, and ideally future controlled studies (e.g., via propensity score matching) should further test the role of timing and pattern of antipsychotics and benzodiazepines prescription in those with first episode of psychosis, while controlling for additional potential confounders.

## Conclusion

In conclusion, we provide the first evidence supporting the notion that delaying antipsychotic prescription for more than 1 week after a FEP may worsen some clinical outcomes. Our results show that early benzodiazepine treatment can be considered with concomitant antipsychotic but not as standalone intervention.

## Data availability statement

The data analyzed in this study is subject to the following licenses/restrictions: the data accessed by CRIS remain within an NHS firewall and governance is provided by a patient-led oversight committee. There is no permission for data sharing. Requests to access these datasets should be directed to Robert Stewart, robert.stewart@kcl.ac.uk, CRIS academic lead.

## Ethics statement

The studies involving human participants were reviewed and approved by the Oxfordshire Research Ethics Committee C; because the data set comprised deidentified data, informed consent was not required. Written informed consent from the participants’ legal guardian/next of kin was not required to participate in this study in accordance with the national legislation and the institutional requirements.

## Author contributions

MS and PF-P designed the study. MA, MS, and TT ran the statistical analyses. All authors drafted, edited, and approved the final version of the manuscript.

## References

[1] Pagsberg AK Tarp S Glintborg D Stenstrøm AD Fink-Jensen A Correll CU Acute antipsychotic treatment of children and adolescents with schizophrenia-spectrum disorders: A systematic review and network meta-analysis. *J Am Acad Child Adolesc Psychiatry.* (2017) 56:191–202. 10.1016/j.jaac.2016.12.013 28219485

[2] Correll CU Cortese S Croatto G Monaco F Krinitski D Arrondo G Efficacy and acceptability of pharmacological, psychosocial, and brain stimulation interventions in children and adolescents with mental disorders: An umbrella review. *World Psychiatry.* (2021) 20:244–75. 10.1002/wps.20881 34002501 PMC8129843

[3] Zhu Y Krause M Huhn M Rothe P Schneider-Thoma J Chaimani A Antipsychotic drugs for the acute treatment of patients with a first episode of schizophrenia: A systematic review with pairwise and network meta-analyses. *Lancet Psychiatry.* (2017) 4:694–705. 10.1016/S2215-0366(17)30270-5 28736102

[4] Huhn M Nikolakopoulou A Schneider-Thoma J Krause M Samara M Peter N Comparative efficacy and tolerability of 32 oral antipsychotics for the acute treatment of adults with multi-episode schizophrenia: A systematic review and network meta-analysis. *Lancet.* (2019) 394:939–51. 10.1016/S0140-6736(19)31135-3 31303314 PMC6891890

[5] Vermeulen JM van Rooijen G van de Kerkhof MPJ Sutterland AL Correll CU de Haan L. Clozapine and long-term mortality risk in patients with schizophrenia: A systematic review and meta-analysis of studies lasting 1.1-12.5 years. *Schizophr Bull.* (2019) 45:315–29. 10.1093/schbul/sby052 29697804 PMC6403051

[6] Correll CU Rubio JM Inczedy-Farkas G Birnbaum ML Kane JM Leucht S. Efficacy of 42 pharmacologic cotreatment strategies added to antipsychotic monotherapy in schizophrenia: Systematic overview and quality appraisal of the meta-analytic evidence. *JAMA Psychiatry.* (2017) 74:675–84. 10.1001/jamapsychiatry.2017.0624 28514486 PMC6584320

[7] National Institute for Health and Care Excellence. *Psychosis and schizophrenia in children and young people: Recognition and management.* London: NICE (2016). 32186837

[8] Nationanal Collaborating Centre for Mental Health. *Schizophrenia: The NICE guideline on core interventions in the treatment and management of schizophrenia in adults in primary and secondary care.* London: Nationanal Collaborating Centre for Mental Health (2010).

[9] Leucht S Leucht C Huhn M Chaimani A Mavridis D Helfer B Sixty Years of placebo-controlled antipsychotic drug trials in acute schizophrenia: Systematic review, bayesian meta-analysis, and meta-regression of efficacy predictors. *Am J Psychiatry.* (2017) 174:927–42. 10.1176/appi.ajp.2017.16121358 28541090

[10] Leucht S Hierl S Kissling W Dold M Davis JM. Putting the efficacy of psychiatric and general medicine medication into perspective: Review of meta-analyses. *Br J Psychiatry.* (2012) 200:97–106. 10.1192/bjp.bp.111.096594 22297588

[11] Fusar-Poli P Estradé A Stanghellini G Venables J Onwumere J Messas G The lived experience of psychosis: A bottom-up review co-written by experts by experience and academics. *World Psychiatry.* (2022) 21:168–88. 10.1002/wps.20959 35524616 PMC9077608

[12] Fusar-Poli P McGorry PD Kane JM. Improving outcomes of first-episode psychosis: An overview. *World Psychiatry.* (2017) 16:251–65. 10.1002/wps.20446 28941089 PMC5608829

[13] McCutcheon RA Pillinger T Efthimiou O Maslej M Mulsant BH Young AH Reappraising the variability of effects of antipsychotic medication in schizophrenia: A meta-analysis. *World Psychiatry.* (2022) 21:287–94. 10.1002/wps.20977 35524614 PMC9077611

[14] Winkelbeiner S Leucht S Kane JM Homan P. Evaluation of differences in individual treatment response in schizophrenia spectrum disorders: A meta-analysis. *JAMA Psychiatry.* (2019) 76:1063–73. 10.1001/jamapsychiatry.2019.1530 31158853 PMC6547253

[15] McCutcheon RA Pillinger T Mizuno Y Montgomery A Pandian H Vano L The efficacy and heterogeneity of antipsychotic response in schizophrenia: A meta-analysis. *Mol Psychiatry.* (2021) 26:1310–20. 10.1038/s41380-019-0502-5 31471576 PMC7610422

[16] American Psychiatric Association. *Diagnostic and statistical manual of mental disorders.* 5th ed. Washington, DC: American Psychiatric Association (2013). 10.1176/appi.books.9780890425596

[17] Guloksuz S Pries L ten Have M de Graaf R van Dorsselaer S Klingenberg B Association of preceding psychosis risk states and non-psychotic mental disorders with incidence of clinical psychosis in the general population: A prospective study in the NEMESIS-2 cohort. *World Psychiatry.* (2020) 19:199–205. 10.1002/wps.20755 32394548 PMC7215054

[18] Fusar-Poli P Nelson B Valmaggia L Yung AR McGuire PK. Comorbid depressive and anxiety disorders in 509 individuals with an at-risk mental state: Impact on psychopathology and transition to psychosis. *Schizophr Bull.* (2014) 40:120–31. 10.1093/schbul/sbs136 23180756 PMC3885287

[19] Mishara AL. Klaus conrad (1905–1961): Delusional mood, psychosis, and beginning schizophrenia. *Schizophr Bull.* (2010) 36(1):9–13. 10.1093/schbul/sbp144 19965934 PMC2800156

[20] Fusar-Poli P Salazar de Pablo G Rajkumar RP López-Díaz Á Malhotra S Heckers S Diagnosis, prognosis, and treatment of brief psychotic episodes: A review and research agenda. *Lancet Psychiatry.* (2022) 9:72–83. 10.1016/S2215-0366(21)00121-8 34856200

[21] World Health Organization [WHO]. *International classification of diseases – 10 (ICD-10).* Geneva: World Health Organization (2019).

[22] Fusar-Poli P Cappucciati M De Micheli A Rutigliano G Bonoldi I Tognin S Diagnostic and prognostic significance of brief limited intermittent psychotic symptoms (BLIPS) in individuals at ultra high risk. *Schizophr Bull.* (2017) 43:48–56. 10.1093/schbul/sbw151 28053130 PMC5216865

[23] Salazar de Pablo G Radua J Pereira J Bonoldi I Arienti V Besana F Probability of transition to psychosis in individuals at clinical high risk: An updated meta-analysis. *JAMA Psychiatry.* (2021) 78:970–8. 10.1001/jamapsychiatry.2021.0830 34259821 PMC8281006

[24] Fusar-Poli P Salazar de Pablo G Correll CU Meyer-Lindenberg A Millan MJ Borgwardt S. Prevention of psychosis: Advances in detection, prognosis, and intervention. *JAMA Psychiatry.* (2020) 77:755–65. 10.1001/jamapsychiatry.2019.4779 32159746

[25] Salazar de Pablo G Catalan A Fusar-Poli P. Clinical validity of DSM-5 attenuated psychosis syndrome: Advances in diagnosis, prognosis, and treatment. *JAMA Psychiatry.* (2020) 77:311–20. 10.1001/jamapsychiatry.2019.3561 31746950

[26] Catalan A Salazar de Pablo G Vaquerizo Serrano J Mosillo P Baldwin H Fernández-Rivas A Annual research review: Prevention of psychosis in adolescents – systematic review and meta-analysis of advances in detection, prognosis and intervention. *J Child Psychol Psychiatry.* (2021) 62:657–73. 10.1111/jcpp.13322 32924144

[27] Salazar de Pablo G Soardo L Cabras A Pereira J Kaur S Besana F Clinical outcomes in individuals at clinical high risk of psychosis who do not transition to psychosis: A meta-analysis. *Epidemiol Psychiatr Sci.* (2022) 31:e9. 10.1017/S2045796021000639 35042573 PMC8786617

[28] Salazar de Pablo G Besana F Arienti V Catalan A Vaquerizo-Serrano J Cabras A Longitudinal outcome of attenuated positive symptoms, negative symptoms, functioning and remission in people at clinical high risk for psychosis: A meta-analysis. *EClinicalMedicine.* (2021) 36:100909. 10.1016/j.eclinm.2021.100909 34189444 PMC8219991

[29] Provenzani U Salazar de Pablo G Arribas M Pillmann F Fusar-Poli P. Clinical outcomes in brief psychotic episodes: A systematic review and meta-analysis. *Epidemiol Psychiatr Sci.* (2021) 30:e71. 10.1017/S2045796021000548 35698876 PMC8581951

[30] Murrie B Lappin J Large M Sara G. Transition of substance-induced, brief, and atypical psychoses to schizophrenia: A systematic review and meta-analysis. *Schizophr Bull.* (2020) 46:505–16. 10.1093/schbul/sbz102 31618428 PMC7147575

[31] Solmi M Murru A Pacchiarotti I Undurraga J Veronese N Fornaro M Safety, tolerability, and risks associated with first-and second-generation antipsychotics: A state-of-the-art clinical review. *Ther Clin Risk Manag.* (2017) 13:757–77. 10.2147/TCRM.S117321 28721057 PMC5499790

[32] Solmi M Fornaro M Ostinelli EG Zangani C Croatto G Monaco F Safety of 80 antidepressants, antipsychotics, anti-attention-deficit/hyperactivity medications and mood stabilizers in children and adolescents with psychiatric disorders: A large scale systematic meta-review of 78 adverse effects. *World Psychiatry.* (2020) 19:214–32. 10.1002/wps.20765 32394557 PMC7215080

[33] Howes OD Whitehurst T Shatalina E Townsend L Onwordi EC Mak TLA The clinical significance of duration of untreated psychosis: An umbrella review and random-effects meta-analysis. *World Psychiatry.* (2021) 20:75–95. 10.1002/wps.20822 33432766 PMC7801839

[34] Fusar-Poli P Van Os J. Lost in transition: Setting the psychosis threshold in prodromal research. *Acta Psychiatr Scand.* (2013) 127:248–52. 10.1111/acps.12028 23136851

[35] Modinos G Allen P Grace AA McGuire P. Translating the MAM model of psychosis to humans. *Trends Neurosci.* (2015) 38:129–38. 10.1016/j.tins.2014.12.005 25554679 PMC4455929

[36] Sonnenschein SF Grace AA. Insights on current and novel antipsychotic mechanisms from the MAM model of schizophrenia. *Neuropharmacology.* (2020) 163:107632. 10.1016/j.neuropharm.2019.05.009 31077730 PMC6842083

[37] Włodarczyk A Szarmach J Cubała WJ Wiglusz MS. Benzodiazepines in combination with antipsychotic drugs for schizophrenia: GABA-ergic targeted therapy. *Psychiatr Danub.* (2017) 29:345–8. 28953788

[38] Benchimol EI Smeeth L Guttmann A Harron K Moher D Peteresen I The REporting of studies conducted using observational routinely-collected health data (RECORD) statement. *PLoS Med.* (2015) 12:1001885. 10.1371/journal.pmed.1001885 26440803 PMC4595218

[39] Fusar-Poli P de Micheli A Patel R Signorini L Miah S Spencer T Real-World clinical outcomes two years after transition to psychosis in individuals at clinical high risk: Electronic health record cohort study. *Schizophr Bull.* (2020) 46:1114–25. 10.1093/schbul/sbaa040 32303767 PMC7505186

[40] Stewart R Soremekun M Perera G Broadbent M Callard F Denis M The South London and Maudsley NHS foundation trust biomedical research centre (SLAM BRC) case register: Development and descriptive data. *BMC Psychiatry.* (2009) 9:51. 10.1186/1471-244X-9-51 19674459 PMC2736946

[41] Perera G Broadbent M Callard F Chang CK Downs J Dutta R Cohort profile of the South London and Maudsley NHS foundation trust biomedical research centre (SLaM BRC) case register: Current status and recent enhancement of an electronic mental health record-derived data resource. *BMJ Open.* (2016) 6:e008721. 10.1136/bmjopen-2015-008721 26932138 PMC4785292

[42] Fusar-Poli P Spencer T De Micheli A Curzi V Nandha S McGuire P. Outreach and support in South-London (OASIS) 2001-2020: Twenty years of early detection, prognosis and preventive care for young people at risk of psychosis. *Eur Neuropsychopharmacol.* (2020) 39:111–22. 10.1016/j.euroneuro.2020.08.002 32921544 PMC7540251

[43] Fusar-Poli P Lai S Di Forti M Iacoponi E Thornicroft G McGuire P Early intervention services for first episode of psychosis in South London and the Maudsley (SLaM): 20 years of care and research for young people. *Front Psychiatry.* (2020) 11:577110. 10.3389/fpsyt.2020.577110 33329115 PMC7732476

[44] Oliver D Wong CMJ Bøg M Jönsson L Kinon BJ Wehnert A Transdiagnostic individualized clinically-based risk calculator for the automatic detection of individuals at-risk and the prediction of psychosis: External replication in 2,430,333 US patients. *Transl Psychiatry.* (2020) 10:1–10. 10.1038/s41398-020-01032-9 33122625 PMC7596040

[45] Irving J Patel R Oliver D Colling C Pritchard M Broadbent M Using natural language processing on electronic health records to enhance detection and prediction of psychosis risk. *Schizophr Bull.* (2021) 47:405–14. 10.1093/schbul/sbaa126 33025017 PMC7965059

[46] Pihlajamaa J Suvisaari J Henriksson M Heilä H Karjalainen E Koskela J The validity of schizophrenia diagnosis in the finnish hospital discharge register: Findings from a 10-year birth cohort sample. *Nord J Psychiatry.* (2008) 62:198–203. 10.1080/08039480801983596 18609031

[47] Uggerby P Østergaard SD Røge R Correll CU Nielsen J. The validity of the schizophrenia diagnosis in the Danish psychiatric central research register is good. *Dan Med J.* (2013) 60:A4578. 23461991

[48] Webb JR Addington J Perkins DO Bearden CE Cadenhead KS Cannon TD Specificity of incident diagnostic outcomes in patients at clinical high risk for psychosis. *Schizophr Bull.* (2015) 41:1066–75. 10.1093/schbul/sbv091 26272875 PMC4535651

[49] Davis KAS Sudlow CLM Hotopf M. Can mental health diagnoses in administrative data be used for research? A systematic review of the accuracy of routinely collected diagnoses. *BMC Psychiatry.* (2016) 16:263. 10.1186/s12888-016-0963-x 27455845 PMC4960739

[50] Fok MLY Stewart R Hayes RD Moran P. The impact of co-morbid personality disorder on use of psychiatric services and involuntary hospitalization in people with severe mental illness. *Soc Psychiatry Psychiatr Epidemiol.* (2014) 49:1631–40. 10.1007/s00127-014-0874-4 24715236

[51] Kaplan EL Meier P. Nonparametric estimation from incomplete observations. *J Am Stat Assoc.* (1958) 53:457–81. 10.1080/01621459.1958.10501452

[52] Greenwood M. *A report on the natural duration of cancer. A report on the natural duration of cancer.* (1926). Available online at: https://www.cabdirect.org/cabdirect/abstract/19272700028 (accessed August 15, 2022).

[53] Simonsen E Friis S Haahr U Johannessen JO Larsen TK Melle I Clinical epidemiologic first-episode psychosis: 1-Year outcome and predictors. *Acta Psychiatr Scand.* (2007) 116:54–61. 10.1111/j.1600-0447.2006.00942.x 17559601

[54] Harrigan SM McGorry PD Krstev H. Does treatment delay in first-episode psychosis really matter? *Psychol Med.* (2003) 33:97–110. 10.1017/S003329170200675X 12537041

[55] Morgan C Lappin J Heslin M Donoghue K Lomas B Reininghaus U Reappraising the long-term course and outcome of psychotic disorders: The AESOP-10 study. *Psychol Med.* (2014) 44:2713–26. 10.1017/S0033291714000282 25066181 PMC4134320

[56] Austin SF Mors O Secher RG Hjorthøj CR Albert N Bertelsen M Predictors of recovery in first episode psychosis: The OPUS cohort at 10year follow-up. *Schizophr Res.* (2013) 150:163–8. 10.1016/j.schres.2013.07.031 23932664

[57] White C Stirling J Hopkins R Morris J Montague L Tantam D Predictors of 10-year outcome of first-episode psychosis. *Psychol Med.* (2009) 39:1447–56. 10.1017/S003329170800514X 19187566

[58] Remschmidt H Theisen F. Early-onset schizophrenia. *Neuropsychobiology.* (2012) 66:63–9. 10.1159/000338548 22797279

[59] Greene W. *Accounting for excess zeros and sample selection in poisson and negative binomial regression models. No. EC-94-10. NYU Working Paper.* New York, NY: New York University (1994).

[60] R Core Team. *R: A language and environment for statistical computing.* Vienna: R Foundation for Statistical Computing (2008).

[61] Spencer E Birchwood M McGovern D. Management of first-episode psychosis. *Adv Psychiatr Treat.* (2001) 7:133–40. 10.1192/apt.7.2.133

[62] Emsley R Rabinowitz J Medori R. Time course for antipsychotic treatment response in first-episode schizophrenia. *AJP.* (2006) 163:743–5. 10.1176/ajp.2006.163.4.743 16585455

[63] Gill KM Lodge DJ Cook JM Aras S Grace AA. A novel α5GABA(A)R-positive allosteric modulator reverses hyperactivation of the dopamine system in the MAM model of schizophrenia. *Neuropsychopharmacology.* (2011) 36:1903–11. 10.1038/npp.2011.76 21562483 PMC3154109

[64] Du Y Grace AA. Loss of parvalbumin in the hippocampus of MAM schizophrenia model rats is attenuated by peripubertal diazepam. *Int J Neuropsychopharmacol.* (2016) 19:yw065. 10.1093/ijnp/pyw065 27432008 PMC5137280

[65] Tan KR Rudolph U Lüscher C. Hooked on benzodiazepines: GABAA receptor subtypes and addiction. *Trends Neurosci.* (2011) 34:188–97. 10.1016/j.tins.2011.01.004 21353710 PMC4020178

[66] Egerton A Modinos G Ferrera D McGuire P. Neuroimaging studies of GABA in schizophrenia: A systematic review with meta-analysis. *Transl Psychiatry.* (2017) 7:e1147–1147. 10.1038/tp.2017.124 28585933 PMC5537645

[67] Damiani S Rutigliano G Fazia T Merlino S Berzuini C Bernardinelli L Developing and validating an individualized clinical prediction model to forecast psychotic recurrence in acute and transient psychotic disorders: Electronic health record cohort study. *Schizophr Bull.* (2021) 47:1695–705. 10.1093/schbul/sbab070 34172999 PMC8530399

[68] Fusar-Poli P Cappucciati M Bonoldi I Hui LMC Rutigliano G Stahl DR Prognosis of brief psychotic episodes: A meta-analysis. *JAMA Psychiatry.* (2016) 73:211–20. 10.1001/jamapsychiatry.2015.2313 26764163

[69] Wang HY Guo WJ Li XJ Tao YJ Meng YJ Wang Q Higher required dosage of antipsychotics to relieve the symptoms of first-onset acute and transient psychotic disorder (ATPD) predicted the subsequent diagnostic transition to schizophrenia: A longitudinal study. *Schizophr Res.* (2018) 193:461–2. 10.1016/j.schres.2017.07.011 28739289

[70] Jackson RG Patel R Jayatilleke N Kolliakou A Ball M Gorrell G Natural language processing to extract symptoms of severe mental illness from clinical text: The clinical record interactive search comprehensive data extraction (CRIS-CODE) project. *BMJ Open.* (2017) 7:e012012. 10.1136/bmjopen-2016-012012 28096249 PMC5253558

[71] Slade M Beck A Bindman J Thornicroft G Wright S. Routine clinical outcome measures for patients with severe mental illness: CANSAS and HoNOS. *Br J Psychiatry.* (1999) 174:404–8. 10.1192/bjp.174.5.404 10616605

[72] Ventura J Hellemann GS Thames AD Koellner V Nuechterlein KH. Symptoms as mediators of the relationship between neurocognition and functional outcome in schizophrenia: A meta-analysis. *Schizophr Res.* (2009) 113:189–99. 10.1016/j.schres.2009.03.035 19628375 PMC2825750

[73] Solmi M Radua J Olivola M Croce E Soardo L Salazar de Pablo G Age at onset of mental disorders worldwide: Large-scale meta-analysis of 192 epidemiological studies. *Mol Psychiatry.* (2022) 27:281–95. 10.1038/s41380-021-01161-7 34079068 PMC8960395

[74] Oliver D Reilly TJ Baccaredda Boy O Petros N Davies C Borgwardt S What causes the onset of psychosis in individuals at clinical high risk? A meta-analysis of risk and protective factors. *Schizophr Bull.* (2020) 46:110–20. 10.1093/schbul/sbz039 31219164 PMC6942149

