# Supplementary material for: Timing of antipsychotics and benzodiazepine initiation during a first episode of psychosis impacts clinical outcomes: Electronic health record cohort study
Source: Front Psychiatry. 2022 Sep 23;13:976035. doi: 10.3389/fpsyt.2022.976035 (PMC9539549; doi:10.3389/fpsyt.2022.976035)
Supplement: Supplementary file 1 [file Data_Sheet_1.DOCX]

**eResults 1***: Sociodemographic and clinical characteristics of subjects prescribed with antipsychotics within versus after one week from FEP diagnosis*

No substantial difference emerged between those prescribed with antipsychotics within one week compared to more than one week from diagnosis on gender, ethnicity or employment. Significant differences were however captured for age, marital status, severity (HONOS), antipsychotic or benzodiazepine as first treatment, type of first antipsychotic molecule, FEP diagnosis cluster as well as follow-up time, as shown in the table below. One-way ANOVA tests were run for continuous variables and chi-squared tests for categorical variables. Significant results are considered when p-value <0.01.

1. **Demographic Characteristics**

| Characteristic | Antipsychotic more than 1 week from diagnosis (N = 575) | Antipsychotic within 1 week from diagnosis (N = 3,908) | p-value |
| --- | --- | --- | --- |
| **Age**; mean (SD) | 24.3 (5.8) | 25.4 (5.5) | **<0.001** |
| Gender |  |  | 0.3 |
| Male | 329.0 (57.2) | 2,330.0 (59.6) |  |
| Female | 246.0 (42.8) | 1,577.0 (40.4) |  |
| (Missing) | 0 (0.0) | 1 (0.0) |  |
| Ethnicity |  |  | 0.027 |
| Caucasian | 267.0 (46.4) | 1,541.0 (39.4) |  |
| Black | 205.0 (35.7) | 1,506.0 (38.5) |  |
| Other/Mixed | 61.0 (10.6) | 484.0 (12.4) |  |
| Asian | 37.0 (6.4) | 296.0 (7.6) |  |
| (Missing) | 5 (0.9) | 81(2.1) |  |
| Employment |  |  | >0.9 |
| Other | 136 (23.7) | 762 (19.5) |  |
| Unemployed | 60 (10.4) | 343 (8.8) |  |
| Student | 35 (6.1) | 186 (4.8) |  |
| Employed | 16 (2.8) | 98 (2.5) |  |
| (Missing) | 328 (57.0) | 2,519 (64.5) |  |
| **Marital status** |  |  | **<0.001** |
| Single | 497.0 (86.4) | 3,124.0 (79.9) |  |
| In a relationship | 27.0 (4.7) | 362.0 (9.3) |  |
| Separated or divorced | 17.0 (3.0) | 110.0 (2.8) |  |
| (Missing) | 34.0 (5.9) | 312.0 (8.0) |  |

1. **Clinical Characteristics**

| Characteristic | Antipsychotic more than 1 week from diagnosis (N = 575) | Antipsychotic within 1 week from diagnosis (N = 3,908) | p-value |
| --- | --- | --- | --- |
| **HONOS (severity)**; mean (SD) | 12.8 (6.4) | 11.2 (6.3) | **<0.001** |
| (Missing) | 96 (16.7) | 552 (14.1) |  |
| **Antipsychotic or benzodiazepine as first treatment; n (%)** |  |  | **<0.001** |
| Antipsychotic before benzodiazepine | 301 (52.3) | 2,643 (67.6) |  |
| Benzodiazepine before antipsychotic | 274 (47.7) | 1,265 (32.4) |  |
| **Type of first antipsychotic molecule; n (%)** |  |  | **<0.001** |
| olanzapine | 194.0 (33.7) | 1,750.0 (44.8) |  |
| risperidone | 154.0 (26.8) | 916.0 (23.4) |  |
| aripiprazole | 86.0 (15.0) | 503.0 (12.9) |  |
| quetiapine | 84.0 (14.6) | 320.0 (8.2) |  |
| haloperidol | 17.0 (3.0) | 186.0 (4.8) |  |
| amisulpiride | 21.0 (3.7) | 119.0 (3.0) |  |
| zuclopenthixol | 7.0 (1.2) | 25.0 (0.6) |  |
| chlorpromazine | 0.0 (0.0) | 24.0 (0.6) |  |
| flupenthixol | 1.0 (0.2) | 22.0 (0.6) |  |
| paliperidone | 3.0 (0.5) | 11.0 (0.3) |  |
| pipotiazine | 2.0 (0.3) | 8.0 (0.2) |  |
| trifluoperazine | 0.0 (0.0) | 9.0 (0.2) |  |
| prochlorperazine | 2.0 (0.3) | 3.0 (0.1) |  |
| sulpiride | 3.0 (0.5) | 2.0 (0.1) |  |
| fluphenazine | 0.0 (0.0) | 4.0 (0.1) |  |
| perphenazine | 0.0 (0.0) | 2.0 (0.1) |  |
| ziprasidone | 0.0 (0.0) | 2.0 (0.1) |  |
| levomepromazine | 0.0 (0.0) | 1.0 (0.0) |  |
| Novorapid | 0.0 (0.0) | 1.0 (0.0) |  |
| NovoRapid FlexPen solution for injection | 1.0 (0.2) | 0.0 (0.0) |  |
| **FEP diagnosis cluster; n (%)** |  |  | **<0.001** |
| Schizophrenia | 287.0 (49.9) | 1,442.0 (36.9) |  |
| Other psychotic disorders | 115.0 (20.0) | 932.0 (23.8) |  |
| Acute and transient psychosis | 80.0 (13.9) | 795.0 (20.3) |  |
| Affective psychosis | 58.0 (10.1) | 556.0 (14.2) |  |
| Substance induced psychosis | 35.0 (6.1) | 183.0 (4.7) |  |
| **Follow-up time (weeks); mean (SD)** | 220.5 (145.3) | 161.3 (144.0) | **<0.001** |
