# Supplementary material for: Timing of antipsychotics and benzodiazepine initiation during a first episode of psychosis impacts clinical outcomes: Electronic health record cohort study
Source: Front Psychiatry. 2022 Sep 23;13:976035. doi: 10.3389/fpsyt.2022.976035 (PMC9539549; doi:10.3389/fpsyt.2022.976035)
Supplement: Supplementary file 2 [file Data_Sheet_2.DOCX]

**eResults 2:** *Sociodemographic and clinical characteristics of subjects prescribed with antipsychotics before (versus after) benzodiazepines at any point*

No substantial difference emerged between those prescribed with antipsychotics before (versus after) benzodiazepines at any point on age, gender, ethnicity, employment, marital status or severity (HONOS). Significant differences were however captured for delay to antipsychotic commencement, type of first antipsychotic molecule, FEP diagnosis cluster as well as follow-up time, as shown in the table below. One-way ANOVA tests were run for continuous variables and chi-squared tests for categorical variables. Significant results are considered when p-value <0.01.

**A. Demographic Characteristics**

| Characteristic | Antipsychotic before benzodiazepine (N = 2,944) | Benzodiazepine before antipsychotic (N = 1,539) | p-value |
| --- | --- | --- | --- |
| Age; mean (SD) | 25.4 (5.5) | 25.0 (5.5) | 0.052 |
| **Gender; n (%)** |  |  | **0.004** |
| Male | 1,792.0 (60.9) | 867.0 (56.3) |  |
| Female | 1,151.0 (39.1) | 672.0 (43.7) |  |
| (Missing) | 1 (0.0) | 0 (0.0) |  |
| Ethnicity; n (%) |  |  | 0.14 |
| Caucasian | 1,178.0 (40.0) | 630.0 (40.9) |  |
| Black | 1,099.0 (37.3) | 612.0 (39.8) |  |
| Other/Mixed | 371.0 (12.6) | 174.0 (11.3) |  |
| Asian | 232.0 (7.9) | 101.0 (6.6) |  |
| (Missing) | 64 (2.2) | 22 (1.4) |  |
| Employment; n (%) |  |  | 0.2 |
| Other | 608 (20.7) | 290 (18.8) |  |
| Unemployed | 276 (9.4) | 127 (8.3) |  |
| Student | 134 (4.6) | 87 (5.7) |  |
| Employed | 72 (2.4) | 42 (2.7) |  |
| (Missing) | 1,854 (63.0) | 993 (64.5) |  |
| Marital status; n (%) |  |  | 0.10 |
| Single | 2,361.0 (80.2) | 1,260.0 (81.9) |  |
| In a relationship | 249.0 (8.5) | 140.0 (9.1) |  |
| Separated or divorced | 92.0 (3.1) | 35.0 (2.3) |  |
| (Missing) | 242.0 (8.2) | 104.0 (6.8) |  |

**B. Clinical Characteristics**

| Characteristic | Antipsychotic before benzodiazepine (N = 2,944) | Benzodiazepine before antipsychotic (N = 1,539) | p-value |
| --- | --- | --- | --- |
| HONOS (severity); mean (SD) | 11.4 (6.3) | 11.4 (6.5) | >0.9 |
| (Missing) | 482 (16.4) | 166 (10.8) |  |
| **Antipsychotic timing; n (%)** |  |  | **<0.001** |
| Antipsychotic within 1 week from diagnosis | 2,643 (90) | 1,265 (82) |  |
| Antipsychotic more than 1 week from diagnosis | 301 (10) | 274 (18) |  |
| **Type of first antipsychotic molecule; n (%)** |  |  | **<0.001** |
| olanzapine | 1,269.0 (43.1) | 675.0 (43.9) |  |
| risperidone | 724.0 (24.6) | 346.0 (22.5) |  |
| aripiprazole | 356.0 (12.1) | 233.0 (15.1) |  |
| quetiapine | 239.0 (8.1) | 165.0 (10.7) |  |
| haloperidol | 148.0 (5.0) | 55.0 (3.6) |  |
| amisulpiride | 101.0 (3.4) | 39.0 (2.5) |  |
| zuclopenthixol | 28.0 (1.0) | 4.0 (0.3) |  |
| chlorpromazine | 19.0 (0.6) | 5.0 (0.3) |  |
| flupenthixol | 16.0 (0.5) | 7.0 (0.5) |  |
| paliperidone | 11.0 (0.4) | 3.0 (0.2) |  |
| pipotiazine | 9.0 (0.3) | 1.0 (0.1) |  |
| trifluoperazine | 7.0 (0.2) | 2.0 (0.1) |  |
| prochlorperazine | 3.0 (0.1) | 2.0 (0.1) |  |
| sulpiride | 4.0 (0.1) | 1.0 (0.1) |  |
| fluphenazine | 4.0 (0.1) | 0.0 (0.0) |  |
| perphenazine | 2.0 (0.1) | 0.0 (0.0) |  |
| ziprasidone | 2.0 (0.1) | 0.0 (0.0) |  |
| levomepromazine | 1.0 (0.0) | 0.0 (0.0) |  |
| Novorapid | 1.0 (0.0) | 0.0 (0.0) |  |
| NovoRapid FlexPen solution for injection | 0.0 (0.0) | 1.0 (0.1) |  |
| **FEP diagnosis cluster; n (%)** |  |  | **<0.001** |
| Schizophrenia | 1,245.0 (42.3) | 484.0 (31.4) |  |
| Other psychotic disorders | 660.0 (22.4) | 387.0 (25.1) |  |
| Acute and transient psychosis | 533.0 (18.1) | 342.0 (22.2) |  |
| Affective psychosis | 381.0 (12.9) | 233.0 (15.1) |  |
| Substance induced psychosis | 125.0 (4.2) | 93.0 (6.0) |  |
| **Follow-up time (weeks); mean (SD)** | 175.6 (148.8) | 156.0 (138.3) | **<0.001** |
