# Supplementary material for: Timing of antipsychotics and benzodiazepine initiation during a first episode of psychosis impacts clinical outcomes: Electronic health record cohort study
Source: Front Psychiatry. 2022 Sep 23;13:976035. doi: 10.3389/fpsyt.2022.976035 (PMC9539549; doi:10.3389/fpsyt.2022.976035)
Supplement: Supplementary file 4 [file Table_2.docx]

**eTable 2.** Clustering and distribution of index ICD-10 non-organic psychosis primary diagnoses

| **Primary index diagnosis (n, %)** | **ICD-10 code** | **n (%)** | **ICD-10 diagnosis name** |
| --- | --- | --- | --- |
| Schizophrenia spectrum psychoses  (1,729, 38.6%) | F20.x, except F20.4, F20.5 | 1,583 (35.3) | Schizophrenia, except Post-schizophrenic depression and residual schizophrenia |
|  | F25.x | 146 (3.3) | Schizoaffective disorders |
| Acute and transient psychotic disorder  (875, 19.5 %) | F23.x | 875 (19.5) | Acute and transient psychotic disorders |
| Affective spectrum psychoses  (614, 13.7%) | F30.2 | 149 (3.3) | Mania with psychotic symptoms |
|  | F31.2/5 | 146 (3.3) | Bipolar affective disorder with psychotic symptoms |
|  | F32.3 | 287 (6.4) | Severe depressive episode with psychotic symptoms |
|  | F33.3 | 32 (0.7) | Recurrent depressive disorder, current episode severe with psychotic symptoms |
| Psychotic disorders due to psychoactive substance abuse  (218, 4.9%) | F10.5 | 9 (0.2) | Psychotic disorder due to use of alcohol |
|  | F11.5 | 2 (0.0) | Psychotic disorder due to use of opioids |
|  | F12.5 | 113 (2.5) | Psychotic disorder due to use of cannabinoids |
|  | F13.5 | 1 (0.0) | Psychotic disorder due to use of sedative or hypnotics |
|  | F14.5 | 12 (0.3) | Psychotic disorder due to use of cocaine |
|  | F15.5 | 7 (0.2) | Psychotic disorder due to use of other stimulants, including caffeine |
|  | F16.5 | 6 (0.1) | Psychotic disorder due to use of hallucinogens |
|  | F19.5 | 68 (1.5) | Psychotic disorder due to use multiple drug use and use of other psychoactive substances |
| Other psychotic disorders  (1,047, 23.3%) | F22.x | 58 (1.3) | Persistent delusional disorders |
|  | F24 | 1 (0.0) | Induced delusional disorder |
|  | F28 | 104 (2.3) | Other nonorganic psychotic disorders |
|  | F29 | 816 (18.2) | Unspecified nonorganic psychosis |
|  | F53.1 | 68 (1.5) | Mental and behavioural disorders associated with the puerperium, not elsewhere classified – Puerperal psychosis NOS |
