# Supplementary material for: Timing of antipsychotics and benzodiazepine initiation during a first episode of psychosis impacts clinical outcomes: Electronic health record cohort study
Source: Front Psychiatry. 2022 Sep 23;13:976035. doi: 10.3389/fpsyt.2022.976035 (PMC9539549; doi:10.3389/fpsyt.2022.976035)
Supplement: Supplementary file 5 [file Table_3.DOCX]

**eTable 3.** Sensitivity analysis A: Adjusted multivariable Cox regression (subjects=3,834, voluntary admissions=1,877, compulsory admission=1,747) to assess the effect of antipsychotic timing on the primary outcome after stratification (risk of voluntary [only] or compulsory [only] psychiatric inpatient admission over 6 years after FEP diagnosis). **Statistically significant results (p<0.01) are shown in bold.**

**Legend.** ATPD, acute and transient psychotic disorder; CI, confidence interval; HONOS, Health Of the Nation Outcome Scales; ICD, Internal Classification of Diseases; HR, hazard ratio

|  | | **Voluntary (only) psychiatric admission** | | | **Compulsory (only) psychiatric admission** | | |
| --- | --- | --- | --- | --- | --- | --- | --- |
| **Factor** | | **HR** | **95%CI** | **P value** | **HR** | **95%CI** | **P value** |
| Antipsychotic more than one week after diagnosis (vs <= one week) | | 1.27 | 1.12-1.44 | **<.001** | 0.94 | 0.82-1.08 | .380 |
| Male sex (vs female) | | 1.09 | 0.99-1.20 | .073 | 1.22 | 1.10-1.34 | **<.001** |
| Age (continuous) | | 0.98 | 0.98-0.99 | **<.001** | 0.99 | 0.98-1.00 | **.004** |
| ICD diagnosis (vs ATPD) | Affective psychosis | 0.77 | 0.65-0.91 | .073 | 0.82 | 0.69-0.97 | .022 |
|  | Other psychotic disorders | 0.89 | 0.78-1.03 | **<.001** | 0.95 | 0.82-1.09 | .440 |
|  | Schizophrenia | 0.99 | 0.87-1.11 | **.002** | 0.94 | 0.83-1.07 | .366 |
|  | Substance-induced psychosis | 1.02 | 0.82-1.27 | .110 | 0.97 | 0.77-1.22 | .765 |
| Severity (HONOS) | | 1.01 | 1.00-1.01 | .087 | 1.02 | 1.01-1.03 | **<.001** |
