# Supplementary material for: Timing of antipsychotics and benzodiazepine initiation during a first episode of psychosis impacts clinical outcomes: Electronic health record cohort study
Source: Front Psychiatry. 2022 Sep 23;13:976035. doi: 10.3389/fpsyt.2022.976035 (PMC9539549; doi:10.3389/fpsyt.2022.976035)
Supplement: Supplementary file 7 [file Table_5.DOCX]

**eTable 5.** Sensitivity analysis A: Adjusted multivariable Cox regression (subjects=3,512, voluntary admissions= 1,645, compulsory admission = 1,559) to assess the effect of treatment patterns within the first week after FEP (combination of antipsychotics with benzodiazepine treatment, antipsychotics alone or benzodiazepine alone [vs no treatment]) on the primary outcome after stratification (risk of voluntary [only] or compulsory [only] psychiatric inpatient admission over 6 years after FEP diagnosis). **Statistically significant results (p<0.01) are shown in bold.**

**Legend.** ATPD, acute and transient psychotic disorder; CI, confidence interval; HONOS, Health Of the Nation Outcome Scales; ICD, Internal Classification of Diseases; HR, hazard ratio

|  | | **Voluntary (only) psychiatric admission** | | | **Compulsory (only) psychiatric admission** | | | |
| --- | --- | --- | --- | --- | --- | --- | --- | --- |
| **Factor** | | **HR** | **95%CI** | **P value** | **HR** | **95%CI** | | **P value** |
| Combination of antipsychotics with benzodiazepine (vs antipsychotics alone) | | 0.87 | 0.78-0.98 | .019 | 0.99 | 0.88-1.12 | | .953 |
| Combination of antipsychotics with benzodiazepine (vs benzodiazepine alone) | | 0.82 | 0.66-1.02 | .075 | 1.22 | 0.95-1.56 | | .118 |
| Male sex (vs female) | | 1.08 | 0.98-1.20 | 0.128 | 1.21 | 1.09-1.34 | | **.0005** |
| Age (continuous) | | 0.98 | 0.97-0.99 | **<.001** | 0.98 | 0.97-0.99 | | .**001** |
| ICD diagnosis (vs ATPD) | Affective psychosis | 0.76 | 0.65-0.92 | **.004** | 0.82 | | 0.69-0.98 | .030 |
|  | Other psychotic disorders | 0.85 | 0.74-0.99 | .031 | 0.91 | | 0.79-1.06 | .230 |
|  | Schizophrenia | 0.96 | 0.84-1.09 | .497 | 0.93 | | 0.81-1.06 | .269 |
|  | Substance-induced psychosis | 0.93 | 0.73-1.18 | .543 | 0.93 | | 0.73-1.18 | .534 |
| Severity (HONOS) | | 1.01 | 1.00-1.01 | .176 | 1.02 | | 1.01-1.03 | **<.001** |
