# Supplementary material for: Timing of antipsychotics and benzodiazepine initiation during a first episode of psychosis impacts clinical outcomes: Electronic health record cohort study
Source: Front Psychiatry. 2022 Sep 23;13:976035. doi: 10.3389/fpsyt.2022.976035 (PMC9539549; doi:10.3389/fpsyt.2022.976035)
Supplement: Supplementary file 8 [file Table_6.DOCX]

**eTable 6.** Sensitivity analysis A: stratification of any psychiatric admission by compulsory (only) or voluntary (only) admission.

Zero-inflation negative binomial regressions to assess the effect of exposure variables (antipsychotic timing, prescribing benzodiazepine before antipsychotics at any point and treatment patterns within first week after diagnosis) on secondary outcomes, adjusted for age, sex, severity and diagnosis. *Reference group; IRR < 1 indicates more favourable effects (fewer days) for AP >1wk (aim 1), BDZ given first (aim 2) or AP+BDZ (aim 3).

**Legend.** IRR: Incidence Rate Ratio, CI-L: 95 % Confidence Interval (Lower), CI-H: 95% Confidence Interval (Higher), AP: antipsychotic, BDZ: benzodiazepine, non-MH: non-mental health, A&E: Accident and Emergency

|  | | Aim 1 | | | | Aim 2 | | | | | Aim3 | | | | | | | |
| --- | --- | --- | --- | --- | --- | --- | --- | --- | --- | --- | --- | --- | --- | --- | --- | --- | --- | --- |
|  | | Antipsychotic timing  (>1 week vs <=1 week after diagnosis) (N=3834) | | | | Prescribing benzodiazepine before antipsychotics (at any point) (N=3834) | | | | | Treatment pattern within first week after diagnosis (AP+BDZ vs AP alone) (N=3512) | | | | Treatment pattern within first week after diagnosis (AP+BDZ vs BDZ alone) (N=3512) | | | |
| Duration of admission (days) | months | IRR | CI-L | CI-H | p-value | | IRR | CI-L | CI-H | p-value | IRR | CI-L | CI-H | p-value | IRR | CI-L | CI-H | p-value |
| Compulsory psychiatric admission | 12 | 1.12 | 0.93 | 1.34 | .244 | | 0.83 | 0.74 | 0.93 | **.002** | **0.74** | **0.63** | **0.87** | **<0.001** | 1.15 | 0.84 | 1.57 | 0.373 |
|  | 24 | 1.35 | 1.12 | 1.62 | **.001** | | 0.77 | 0.68 | 0.87 | **<.001** | **0.77** | **0.65** | **0.90** | **0.001** | 1.03 | 0.75 | 1.41 | 0.871 |
|  | 36 | 1.30 | 1.09 | 1.56 | **.004** | | 0.78 | 0.69 | 0.88 | **<.001** | 0.85 | 0.73 | 1.00 | 0.044 | 1.25 | 0.91 | 1.71 | 0.174 |
|  | 48 | 1.34 | 1.12 | 1.61 | **.001** | | 0.77 | 0.69 | 0.87 | **<.001** | 0.83 | 0.71 | 0.97 | 0.022 | 1.13 | 0.82 | 1.54 | 0.449 |
|  | 60 | 1.28 | 1.07 | 1.53 | **.008** | | 0.81 | 0.72 | 0.92 | **.001** | 0.88 | 0.75 | 1.03 | 0.102 | 1.27 | 0.92 | 1.76 | 0.151 |
|  | 72 | 1.27 | 1.05 | 1.53 | .013 | | 0.83 | 0.73 | 0.94 | **.003** | 0.92 | 0.78 | 1.08 | 0.296 | 1.31 | 0.93 | 1.83 | 0.118 |
| Voluntary psychiatric admission | 12 | 1.48 | 1.22 | 1.81 | **<.001** | | 0.85 | 0.74 | 0.97 | .019 | **0.76** | **0.64** | **0.92** | **0.005** | 0.85 | 0.63 | 1.16 | 0.320 |
|  | 24 | 1.46 | 1.20 | 1.77 | **<.001** | | 0.79 | 0.69 | 0.90 | **<.001** | **0.78** | **9.64** | **0.91** | **0.003** | 0.96 | 0.71 | 1.31 | 0.804 |
|  | 36 | 1.36 | 1.13 | 1.63 | **.001** | | 0.76 | 0.67 | 0.86 | **<.001** | **0.80** | **0.68** | **0.94** | **0.007** | 1.01 | 0.75 | 1.36 | 0.942 |
|  | 48 | 1.38 | 1.14 | 1.68 | **.001** | | 0.71 | 0.62 | 0.82 | **<.001** | 0.81 | 0.68 | 0.96 | 0.016 | 1.14 | 0.83 | 1.56 | 0.420 |
|  | 60 | 1.36 | 1.13 | 1.64 | **.001** | | 0.70 | 0.61 | 0.79 | **<.001** | 0.82 | 0.70 | 0.87 | 0.021 | 1.07 | 0.78 | 1.46 | 0.667 |
|  | 72 | 1.45 | 1.20 | 1.75 | **<.001** | | 0.69 | 0.60 | 0.79 | **<.001** | 0.83 | 0.70 | 0.98 | 0.026 | 1.06 | 0.77 | 1.45 | 0.714 |
