# Supplementary material for: Timing of antipsychotics and benzodiazepine initiation during a first episode of psychosis impacts clinical outcomes: Electronic health record cohort study
Source: Front Psychiatry. 2022 Sep 23;13:976035. doi: 10.3389/fpsyt.2022.976035 (PMC9539549; doi:10.3389/fpsyt.2022.976035)
Supplement: Supplementary file 9 [file Table_7.DOCX]

**eTable 7.** Sensitivity analysis B: Adjusted multivariable Cox regression results after stratification of primary outcome by follow-up year (1, 2, 3, 4, 5) after FEP diagnosis. The effect of (A) antipsychotic timing, (B) prescribing benzodiazepine before antipsychotics, and (C) treatment patterns within the first week after FEP, was tested on the primary outcome (risk of any psychiatric inpatient admission), every year up to 5 years after FEP diagnosis. **Statistically significant results (p<0.01) are shown in bold**

**Legend.** ATPD, acute and transient psychotic disorder; CI, confidence interval; HONOS, Health Of the Nation Outcome Scales; ICD, Internal Classification of Diseases; HR, hazard ratio

**A** Antipsychotic more than one week after diagnosis (vs <= one week)

| **Outcome** | **Admissions** | **HR** | **95%CI** | **P value** |
| --- | --- | --- | --- | --- |
| 1 year | 1,129/1,783 | 1.08 | 0.91-1.27 | .381 |
| 2 years | 1,478/2,431 | 1.29 | 1.11-1.49 | **<.001** |
| 3 years | 1,671/2,835 | 1.31 | 1.14-1.51 | **<.001** |
| 4 years | 1,804/3,140 | 1.06 | 0.93-1.21 | .396 |
| 5 years | 1,904/3,345 | 1.09 | 0.96-1.24 | .193 |

**B** Prescribing benzodiazepine before (vs after) antipsychotics (at any point)

| **Outcome** | **Admissions** | **HR** | **95%CI** | **P value** |
| --- | --- | --- | --- | --- |
| 1 year | 1,129/1,783 | 0.88 | 0.78-1.00 | .048 |
| 2 years | 1,478/2,431 | 0.95 | 0.85-1.06 | .360 |
| 3 years | 1,671/2,835 | 0.93 | 0.84-1.03 | .162 |
| 4 years | 1,804/3,140 | 0.95 | 0.86-1.22 | .312 |
| 5 years | 1,904/3,345 | 0.98 | 0.89-1.07 | .616 |

**C)** Combination of antipsychotics with benzodiazepine treatment within first week after FEP

|  | | Combination vs antipsychotics alone | | | Combination vs benzodiazepine alone | | |
| --- | --- | --- | --- | --- | --- | --- | --- |
| **Outcome** | **Admissions** | **HR** | **95%CI** | **P value** | **HR** | **95%CI** | **P value** |
| 1 year | 1,007/1,651 | 0.76 | 0.65-0.89 | **<.001** | 1.00 | 0.76-1.33 | .979 |
| 2 years | 1,327/2,259 | 0.80 | 0.70-0.92 | **.001** | 0.83 | 0.64-1.07 | .145 |
| 3 years | 1,504/2,640 | 0.83 | 0.73-0.94 | **.004** | 0.91 | 0.71-1.15 | .427 |
| 4 years | 1,614/2,907 | 0.88 | 0.78-0.99 | .031 | 1.11 | 0.88-1.40 | .397 |
| 5 year | 1,701/3,086 | 0.90 | 0.80-1.01 | .083 | 0.97 | 0.77-1.22 | .805 |
