# Supplementary material for: Timing of antipsychotics and benzodiazepine initiation during a first episode of psychosis impacts clinical outcomes: Electronic health record cohort study
Source: Front Psychiatry. 2022 Sep 23;13:976035. doi: 10.3389/fpsyt.2022.976035 (PMC9539549; doi:10.3389/fpsyt.2022.976035)
Supplement: Supplementary file 10 [file Table_8.docx]

**eTable 8.** Sensitivity analysis B: Subset of patients with F23 diagnostic classification (N = 783).

Zero-inflation negative binomial regressions to assess the effect of exposure variables (antipsychotic timing, prescribing benzodiazepine before antipsychotics at any point and treatment patterns within first week after diagnosis) on secondary outcomes, adjusted for age, sex, severity and diagnosis. *Reference group; IRR < 1 indicates more favourable effects (fewer days) for AP >1wk (aim 1), BDZ given first (aim 2) or AP+BDZ (aim 3). **Statistically significant results (p<0.01) are shown in bold.**

**Legend.** IRR: Incidence Rate Ratio, CI-L: 95 % Confidence Interval (Lower), CI-H: 95% Confidence Interval (Higher), AP: antipsychotic, BDZ: benzodiazepine, non-MH: non-mental health, A&E: Accident and Emergency

|  | | Aim 1 | | | | Aim 2 | | | | Aim 3 | | | | | | | |
| --- | --- | --- | --- | --- | --- | --- | --- | --- | --- | --- | --- | --- | --- | --- | --- | --- | --- |
|  | | Antipsychotic timing  (>1 wk vs <=1 wk after diagnosis) | | | | Prescribing benzodiazepine before antipsychotics (at any point) | | | | Treatment pattern within first week after diagnosis (AP+BDZ vs AP alone) | | | | Treatment pattern within first week after diagnosis (AP+BDZ vs BDZ alone) | | | |
| Duration of admission (days) | months | IRR | CI-L | CI_H | p-value | IRR | CI-L | CI-H | p-value | IRR | CI-L | CI-H | p-value | IRR | CI-L | CI-H | p-value |
| Any psychiatric admission | 12 | 1.23 | 0.85 | 1.78 | .278 | 0.87 | 0.70 | 1.08 | .215 | 0.91 | 0.67 | 1.24 | 0.559 | 1.12 | 0.64 | 1.96 | 0.682 |
|  | 24 | 1.32 | 0.90 | 1.94 | .152 | 0.80 | 0.64 | 1.00 | .050 | 0.93 | 0.69 | 1.26 | 0.645 | 1.11 | 0.64 | 1.93 | 0.717 |
|  | 36 | 1.21 | 0.83 | 1.76 | .319 | 0.81 | 0.65 | 1.01 | .067 | 1.04 | 0.77 | 1.40 | 0.786 | 1.28 | 0.72 | 2.27 | 0.393 |
|  | 48 | 1.26 | 0.86 | 1.83 | .239 | 0.82 | 0.66 | 1.02 | .079 | 1.03 | 0.76 | 1.38 | 0.858 | 1.28 | 0.73 | 2.24 | 0.396 |
|  | 60 | 1.18 | 0.80 | 1.73 | .401 | 0.87 | 0.69 | 1.09 | .216 | 1.14 | 0.85 | 1.54 | 0.379 | 1.44 | 0.80 | 2.60 | 0.225 |
|  | 72 | 1.19 | 0.81 | 1.75 | .386 | 0.88 | 0.70 | 1.10 | .269 | 1.19 | 0.88 | 1.60 | 0.258 | 1.52 | 0.83 | 2.77 | 0.176 |
| Medical non-MH admission | 12 | 0.70 | 0.18 | 2.80 | .618 | 1.88 | 0.86 | 4.19 | .111 | 2.86 | 1.78 | 4.60 | **<.001** | 0.68 | 0.09 | 4.97 | 0.704 |
|  | 24 | 0.55 | 0.17 | 1.75 | .312 | 2.37 | 1.25 | 4.47 | **.008** | 2.16 | 1.49 | 3.12 | <.001 | 0.85 | 0.16 | 4.52 | 0.845 |
|  | 36 | 0.47 | 0.19 | 1.18 | .110 | 1.46 | 0.87 | 2.44 | .149 | 1.42 | 1.04 | 1.92 | 0.026 | 0.93 | 0.24 | 3.58 | 0.921 |
|  | 48 | 0.41 | 0.15 | 1.13 | .086 | 1.49 | 0.85 | 2.59 | .162 | 1.77 | 1.27 | 2.45 | **<.001** | 1.09 | 0.25 | 4.75 | 0.908 |
|  | 60 | 0.43 | 0.18 | 1.05 | .064 | 1.23 | 0.74 | 2.04 | .416 | 1.37 | 1.02 | 1.85 | 0.035 | 1.04 | 0.28 | 3.83 | 0.956 |
|  | 72 | 0.36 | 0.15 | 0.86 | .022 | 1.30 | 0.76 | 2.22 | .344 | 1.42 | 2.36 | 1.90 | 0.018 | 1.58 | 0.41 | 6.03 | 0.503 |
| A&E admission | 12 | 1.17 | 0.76 | 1.80 | .471 | 0.91 | 0.70 | 1.19 | .491 | 0.80 | 0.53 | 1.22 | 0.298 | 0.69 | 0.36 | 1.31 | 0.255 |
|  | 24 | 1.12 | 0.76 | 1.67 | .568 | 0.92 | 0.72 | 1.16 | .459 | 0.95 | 0.68 | 1.32 | 0.748 | 0.64 | 0.35 | 1.17 | 0.145 |
|  | 36 | 1.10 | 0.74 | 1.62 | .645 | 0.94 | 0.75 | 1.17 | .557 | 0.92 | 0.67 | 1.26 | 0.590 | 0.68 | 0.37 | 1.25 | 0.216 |
|  | 48 | 0.92 | 0.63 | 1.32 | .638 | 0.89 | 0.72 | 1.12 | .322 | 0.86 | 0.64 | 1.16 | 0.336 | 0.78 | 0.44 | 1.40 | 0.403 |
|  | 60 | 0.90 | 0.61 | 1.34 | .611 | 0.89 | 0.72 | 1.12 | .318 | 0.86 | 0.64 | 1.14 | 0.292 | 0.85 | 0.48 | 1.52 | 0.591 |
|  | 72 | 0.90 | 0.61 | 1.33 | .599 | 0.88 | 0.71 | 1.10 | .255 | 0.88 | 0.60 | 1.29 | 0.519 | 0.89 | 0.50 | 1.58 | 0.688 |
